# Faster and smaller: Heterochronic shift in gene expression leads to larval onset of spermatogenesis and miniaturized males

**DOI:** 10.1016/j.isci.2026.117511

**Published:** 2026-09-17

**Authors:** Alice Rouan, Madeleine E. Aase-Remedios, Flora Lind-Rasmussen, Norio Miyamoto, Katrine Worsaae

**Affiliations:** 1 Marine Biological Section, Department of Biology, University of Copenhagen, Universitetsparken 4, 2100 Copenhagen Ø, Denmark; 2 X-STAR, Japan Agency for Marine-Earth Science and Technology, Yokosuka, Japan

**Keywords:** larval testis, germ cells, progenesis, dwarf males, sexual dimorphism

## Abstract

Heterochrony, a change in the timing of developmental events, is suggested as the prevailing evolutionary route to an underdeveloped or pedomorphic body plan. Many microscopic animal lineages (meiofauna) are hypothesized to descend from macroscopic ancestors, but direct evidence linking miniaturization to heterochronic shifts in development is scarce. Here, we investigate the molecular developmental basis of the dimorphic annelid *Osedax japonicus*, which has bone-eating macroscopic females and microscopic, pedomorphic males. We find that males start their sexual maturation extremely early, with testes already developing in the larval stage, and cease their somatic growth soon after metamorphosis. Heterochronic shifts in gene expression explain this male pedomorphosis through early onset of germline development, sexual determination, and maturation in concert with early offset of somatic growth. Our findings provide direct developmental evidence for how heterochrony can drive extreme miniaturization, shedding new light on the evolutionary origins of microscopic animal lineages.

## Highlights

• Sexual maturation begins pre-metamorphosis in dwarf male *Osedax japonicus*
• Rapid sexual maturation co-occurs with early somatic growth arrest in progenetic males
• Heterochronic shifts in gene expression extend to development and morphogenesis
• FoxO signaling may contribute to somatic growth arrest in dwarf males

## Graphical abstract

**Figure:**
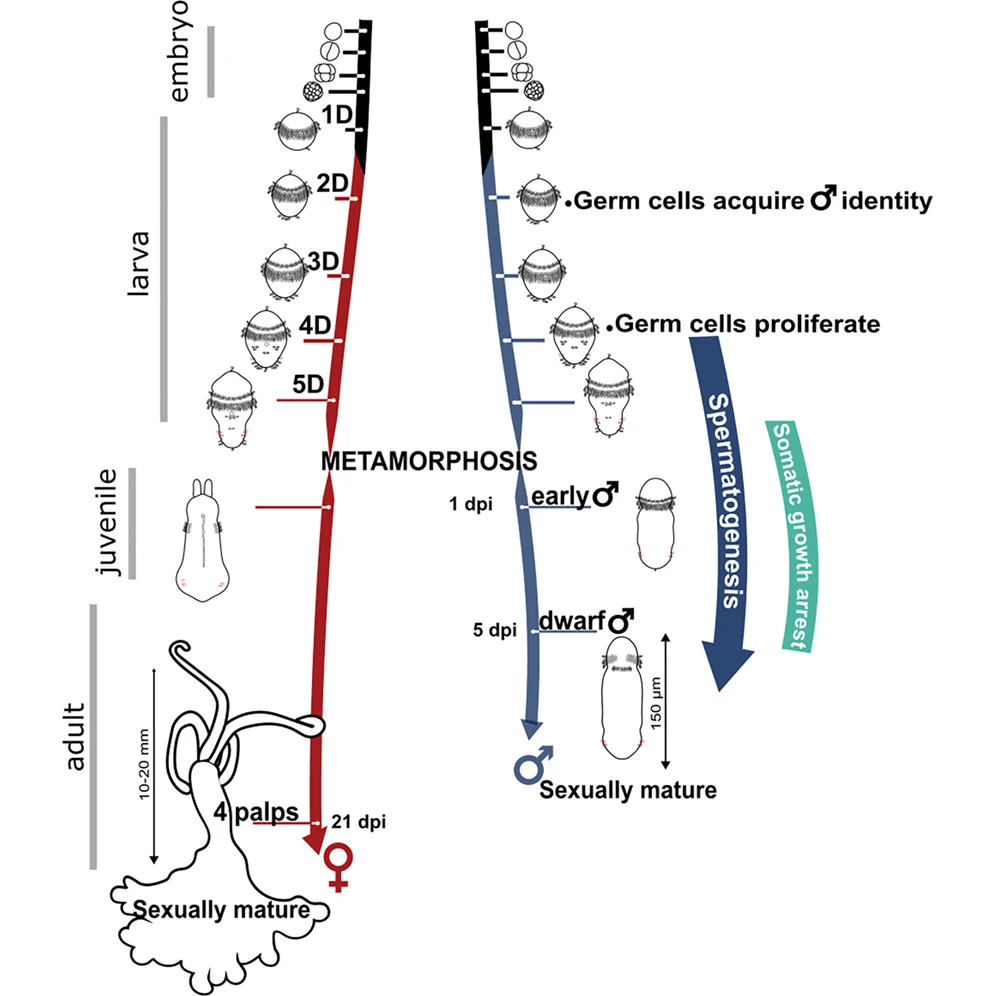

## Teaser

Evolutionary Developmental Biology; Genomics

## Introduction

The temporal coordination of developmental programs is essential for ensuring proper organismal formation and the successful onset of sexual maturation. Although a change of ontogenetic temporality, called heterochrony, can lead to malformation, it is also seen as a driving force of evolution.^1^^,^^2^^,^^3^^,^^4^ Yet, few studies have documented heterochronic shifts empirically.^5^^,^^6^^,^^7^^,^^8^^,^^9^^,^^10^ Heterochronic shifts are suggested as the prevailing evolutionary route to an underdeveloped or pedomorphic body plan.^1^^,^^2^^,^^3^^,^^11^ This can result either from a slower rate (neoteny), a late onset (post-displacement), or an early offset (progenesis) of development.^2^^,^^3^ Theoretically, an early global offset of somatic growth (progenesis) would lead to a miniaturized, pedomorphic body plan of the descendant compared to the ancestor. Moreover, multiple processes may be involved, for instance, progenesis has been proposed to be initiated by an accelerated or early onset of sexual maturation causing an early offset in somatic growth (e.g., Denoël and Joly,^5^ Worsaae et al.,^10^ and Westheide^12^). The extent to which microscopic, miniaturized lineages reflect such heterochronic processes has rarely been documented, in part due to the vast evolutionary distance to comparative macroscopic lineages, so the underlying genetic mechanisms remain largely unknown.

**Figure 1:**
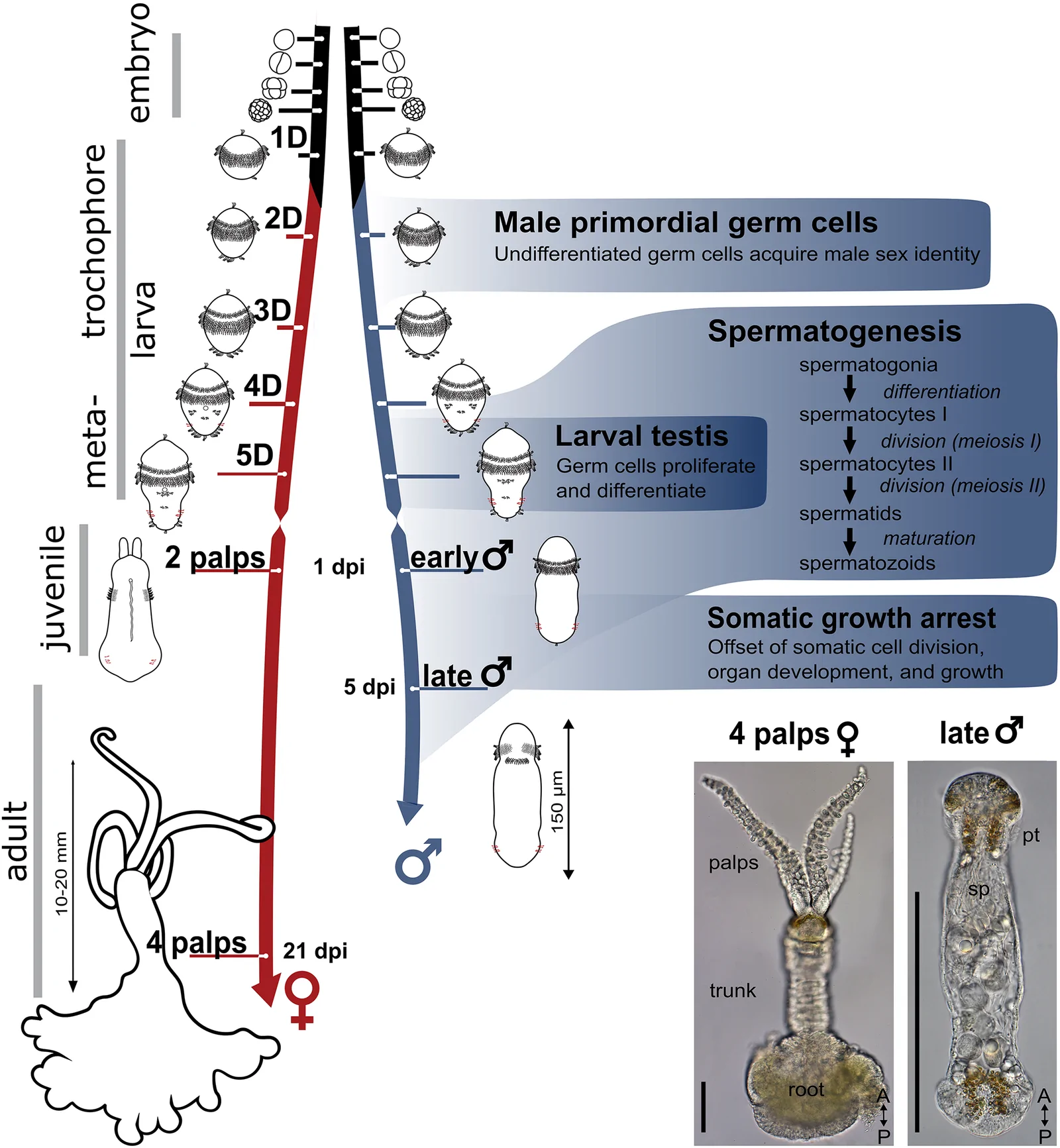
*Osedax japonicus* male dwarfism is a result of progenesis comprising an early onset of spermatogenesis and an early offset of somatic growth Life cycle of *O. japonicus*. From the 2-day larval stage, sexes can be distinguished by gene expression signatures. From the 4-day larval stage, sexes can be distinguished morphologically by the presence, in females, or absence, in males, of a ciliated gut. After 5 days, the metatrochophore larva is competent for metamorphosis. The juveniles grow at different rates; males reach sexual maturity/adulthood at 5-day post induction (dpi) of metamorphosis while it takes 3 weeks (21 dpi) for females. Adult male is around 100 times smaller than the adult female. Scale bars are 100 μm. A, anterior; P, posterior; pt, prototroch; sp, spermatozoid.

Sexually dimorphic *Osedax* (Annelida) present a unique opportunity to examine the mechanisms of pedomorphosis (progenesis in particular) through comparative developmental studies of the miniaturized, pedomorphic male relative to the macroscopic female of the same species.^10^^,^^13^^,^^14^^,^^15^ *Osedax* females feed on bones through symbiosis with heterotrophic bacteria, while the non-feeding dwarf males aggregate in harems on the female trunk.^13^^,^^16^^,^^17^^,^^18^ *Osedax* male adults (except for *O. priapus*^19^) largely resemble the swimming larval stage (metatrochophore) and are at least 100 times smaller in body size than females^10^^,^^14^^,^^15^^,^^20^^,^^21^^,^^22^^,^^23^ (Figure 1). *Osedax japonicus* dwarf males undergo a very fast sexual maturation, resulting in the production of mature spermatozoids only a few days after metamorphosis, and synchronously cease their somatic development. In contrast, it takes 3 weeks following metamorphosis for female *O. japonicus* to reach sexual maturity and full somatic development^10^^,^^15^^,^^21^ (Figure 1). Yet, the molecular mechanisms underlying this dimorphism are still unknown. The recent staging of *Osedax* development^10^ and individual transcriptomes from each life stage^23^^,^^24^ have allowed for a range of comparisons and the discovery of sexually differentiated larvae.^24^ With the transcriptomic and morphological profiling of male and female larvae, we can now investigate the molecular basis of heterochrony and pedomorphosis throughout the entire development of *O. japonicus*.

In sexually reproducing animals, sexual maturation is preceded by segregation of primordial germ cells (PGCs) that will later produce the gametes upon sexual determination. Conserved genes such as *nanos*,^25^ *vasa*^26^ (or *ddx4*), and *piwi*^27^^,^^28^ are involved in germline segregation.^29^ Sexual determination, when genetically controlled, can be specified by the expression of conserved transcription factors such as DMRTs.^30^^,^^31^ *Dmrt1* or *Dmrt1L* are male-specific determinants in mollusks, platyhelminthes, and the annelid *Platynereis dumerilii*.^32^^,^^33^^,^^34^ Homologs of the human *Sox9*, such as *SoxH* or *Sox100B* in *Drosophila melanogaster*, can also play a determining role in male determination,^35^ while *FoxL2* induces the female phenotype in mollusks.^31^^,^^36^ The acquisition of germline sexual identity often coincides with gonad development. In most invertebrates, gonad development occurs in the juvenile stage, post-metamorphosis for indirect developers or post-hatching for direct developers, whereas only a few species, such as the fruit fly *D. melanogaster*, form an embryonic gonad.^37^

During male sexual maturation, the germ cells undergo spermatogenesis to produce viable gametes. Spermatogenesis encompasses three phases: first, the PGCs differentiate into proliferating spermatogonia, which will thereafter differentiate into spermatocytes I (proliferation phase). Spermatocytes undergo two rounds of meiosis (meiotic phase) to produce early spermatids, which will then differentiate into late spermatids and then spermatozoa through the process of spermiogenesis (differentiation phase).^38^ Various genes can mark the distinct stages of spermatogenesis, such as the transcription factors *Myc*, *SoxH*, or *FoxJ1*, expressed in the testis and spermatocytes II in two mollusks^36^^,^^39^; the testis-specific serine kinase (*TSSK*) family^39^; and the tubulin beta (TBB) genes, involved in flagellum formation.^39^^,^^40^^,^^41^

To identify genetic signatures of pedomorphosis, we used the annelid *Osedax japonicus*, with macroscopic females and microscopic, pedomorphic males. We investigated the timing of male sexual maturation by tracing the onset of spermatogenesis throughout development, combining cell proliferation assays, *in situ* hybridization chain reaction (HCR) of known markers (e.g., *piwi* and *TBB*), and differential gene expression (DGE) analysis. We show that pedomorphic males already initiate gonad development and spermatogenesis during larval stages. We confirm the morphologically described early somatic growth arrest, a defining aspect of progenesis, using a cell proliferation assay. We also describe new genes that undergo shifts in expression consistent with early offset and/or early onset in males, revealing functions related to pedomorphosis that encompasses sexual maturation.

## Results

### A larval reproductive tissue develops pre-metamorphosis

**Figure 2:**
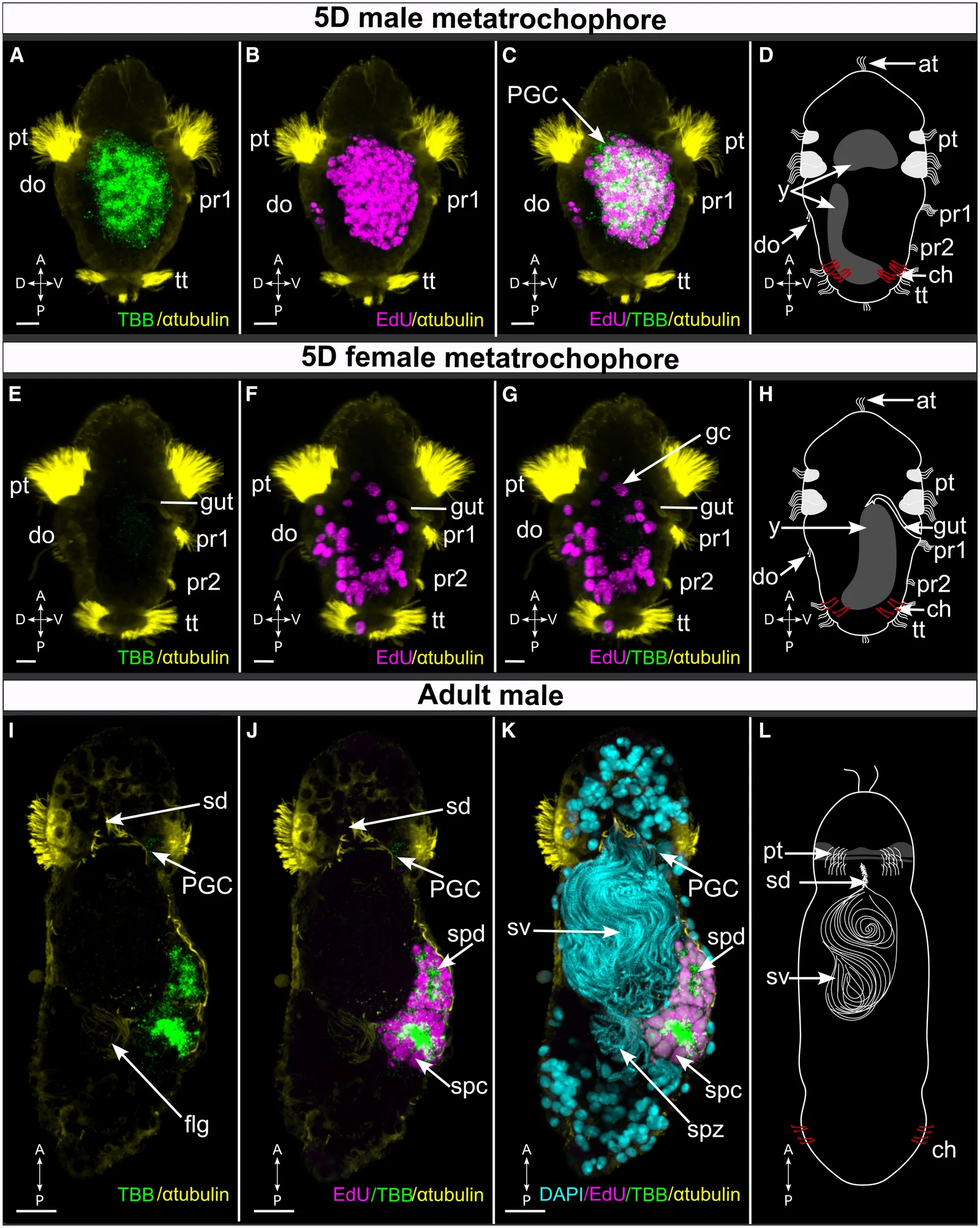
Pre-metamorphic cell proliferation in male larvae germ cells (A–D) Lateral view of central maximum z-projection (MIP) sections and drawing of a 5-day male-competent larva showing the proliferation of mesodermal *TBB*-positive cells. (E–H) Lateral view of MIP sections and drawing of a 5-day female larva showing scattered cell proliferation and *TBB* expression in gut cells. (I–L) Dorsal view of MIP sections and drawing of adult males showing the expression of *TBB* in EdU-positive spermatocytes and spermatids. Expression of tubulin beta (*TBB*, green) in proliferative cells (magenta) and acetylated α-tubulin-like immunoreactivity (LIR) (yellow). Scale bars are 10 μm. A, anterior; at, apical tuft; ch, chaetae; D, dorsal; do, dorsal organ; flg, flagellum; gc, gut cell; PGC, primordial germ cell; P, posterior; pt, prototroch; pr1/2, paratroch 1/2; sd, seminal duct; spc, spermatocyte; spd, spermatid; spz, spermatozoid; sv, seminal vesicle; tt, telotroch; V, ventral; y, yolk.

*Osedax* larvae were recently found to be morphologically sexually differentiated from 4-day larval stages by the presence of a ciliated transient gut, visualized with acetylated-α-tubulin-like immunoreactivity (LIR), in females or an absence in males^24^ (Figures 1, 2D, and 2H). To identify male gonads, we coupled an EdU proliferation assay with whole-mount *in situ* HCR to localize the expression of *TBB*, a main component of the flagellum axoneme^38^ and a marker of male germ cells,^40^ as well as a general marker of ciliated cells. In male larvae, *TBB* is distinctly expressed in a large, central group of proliferating mesodermal cells that are absent in females (Figures 2C versus 2G). Indeed, *TBB* expression marks the male germline in *O. japonicus*, as it is detected in adult males in dividing germ cells undergoing spermatogenesis, such as spermatocytes and spermatids (Figures 2I–2K).

Similar to the 5-day male larva (Figures 2A and 2C), *TBB* signal is also found in large non-dividing PGCs in the anteriormost part of the adult’s first segment (Figures 2J and 2K). In the male larva, both the mesodermal and central positioning of these male specific cells, and the similarity of their *TBB*^+^ signal to that of the adult male germ cells (Figures 2C and 2K), suggest the onset of male reproductive tissue development.

The yolk in the male larva is restricted to the dorsal and anterior part by the proliferating germ cells, while positioned medio-ventrally in the female larva (Figures 2D versus 2H). Besides from the germline proliferation in male larvae, both sexes of 5-day larvae show a similar somatic proliferation in the dorsal sensory organ (Figures 2B and 2F), while only female larvae exhibit a sparse number of peripherally proliferating cells (Figure 2F).

### PGCs acquire sexual identity early in male larval development

**Figure 3:**
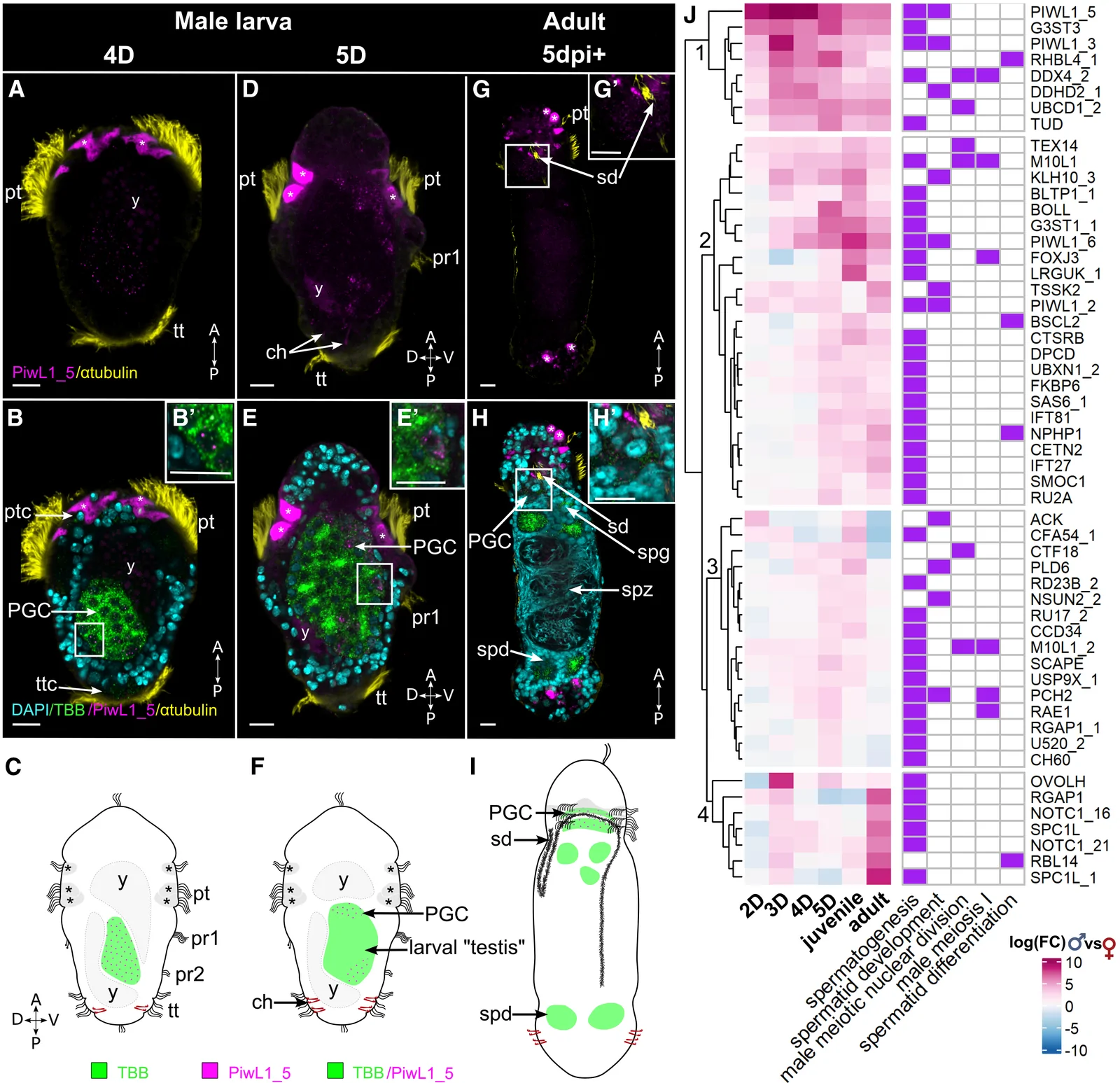
Early acquisition of sexual identity of primordial germ cells and transcriptomic signatures of spermatogenesis (A–C) Dorsal view of maximum z-intensity projection (MIP) sections and drawing of a 4-day male larva showing a co-expression of *TBB* (green) and *PiwiL1_5* (magenta) coupled with nuclei staining (DAPI, cyan) and acetylated alpha-tubulin-like immunoreactivity (LIR) (yellow). (D–F) Lateral view of MIP middle sections and drawing of 5-day male larva showing the mesodermal expression of *TBB* and the restricted co-expression of *PiwiL1_5* to the primordial germ cells (PGCs). (G–I) Mid-dorsal view of MIP sections and drawing of an adult male showing the expression of *TBB* in spermatogonia and spermatocytes and *PiwiL1_5* anterior restriction to the PGC. (J) Heatmap representing the log(foldchange) (logFC) of Gene Ontology (GO) term “male gamete formation” genealogy genes (purple), overexpressed in at least one male larval stage. Scale bars are 10 μm. A, anterior; ch, chaetae; D, dorsal; P, posterior; pt, prototroch; pr1/2, paratroch 1/2; sd, seminal duct; spd, spermatid; spg, spermatogonia; spz, spermatozoid; tt, telotroch; ttc, telotroch cell; V, ventral; y, yolk; ∗ shows non-specific signal of prototroch gland cells.

To confirm the establishment of germ cell sexual identity, required for the development of a larval reproductive tissue, we investigated the expression of germline markers (*nanos*, *Piwi*, and *Piwi1-*like) (Table S1). Among the seven male-specific genes selected (see STAR Methods), only the probes designed against the germline marker *PiwiL1_5* displayed a distinct signal and were investigated in combination with the male gamete marker *TBB*. A distinct co-expression of *TBB* and *PiwiL1_5* is found in the central group of germline cells observed only in male (Figure 3A) and not in female larvae. A similar co-expression pattern in mesodermal cells is observed from the 2- to 4-day male larval stage (Figures 3A–3C and S1). In the 4-day male larvae, a larger group of mesodermal cells expresses both *TBB* and the male-specific germline marker *PiwiL1_5* (Figures 3B and 3B′). In the 5-day male larvae, *PiwiL1_5* expression becomes restricted to the anterior and posterior subclusters of the *TBB*^+^ cells (Figures 3D–3E′). This suggests that a population of PGCs has now differentiated, supporting the development of the larval reproductive tissue (Figure 2). In the adult male, *PiwiL1_5*-positive cells are restricted to the anterior part of the first segment, consistent with the described position of PGCs (Figures 2 and 3H–3H′). *TBB* is also expressed in somatic ciliated cells forming the trochs in both sexes (paratroch 1 and 2, telotroch, and prototroch) (Figures 3B and S1). Additionally, a non-specific signal can be observed in the autofluorescent chaetae in the 5-day larvae and adults (Figure 3D), in the pigmented prototroch gland cells, and in yolk cell granules (Figure 3A).

### Early onset of expression of genes involved in spermatogenesis

To further characterize the genetic onset of sexual maturation in males compared to females, we performed DGE analysis between sexes^23^^,^^24^ (Figure S2; Tables S2 and S3). Among the annotated genes we found overexpressed in at least one male larval stage (logFC > 2), we selected those annotated with the “male gamete generation” Gene Ontology (GO) term (GO:0048232) and its descendants (Figure 3J; Table S3). The genes in the top group of the heatmap display the earliest overexpression in male, either starting in the 2- or 3-day larval stage (Figure 3J). They include known germline markers *PiwiL1_5*, *PiwiL1_6*, *Ddx4* (*vasa*), and *Tud* (*tudor*) and are associated with GO terms ranging from spermatogenesis to spermatid differentiation (Figure 3J). *PiwiL1_5* is most highly overexpressed in males compared to females at the 2-day larval stage (logFC = 9.4, adjusted *p* value = 0.02) and gradually decreases from the 5-day larvae onward, matching the expression patterns observed by HCR (Figures 3A–3J; Table S2). The second group contains genes expressed mainly in the 5-day larvae, early and late males, associated with spermatogenesis, spermatid development (*TSSK2*, *KLH10_3*, *PiwiL1_2*, and *PiwiL1_6*), male meiotic nuclear division (*ML10L1* and *Tex14*), male meiosis (*M10L1* and *FoxJ3*), and spermatid differentiation (*BSCL2* and *NPHP1*) (Figure 3J). The third group combines genes with a significant overexpression in 5-day larvae and early males, with some being associated with male meiosis GO term (*PCH2*, *RAE1*, and *M10L1_2*) (Figure 3J). More specifically, *RAE1* is expressed in *D. melanogaster* spermatocytes I, being necessary for nuclear integrity in those cells,^42^ whereas the ATPase *PCH2* is involved in the generation of spermatocytes II through meiosis I in *Caenorhabditis elegans*.^43^ The fourth group contains genes overexpressed in the early 3-day larval stage and again in the late male, suggesting an involvement in distinct pathways (Figure 3J). Other genes, only overexpressed in post-metamorphosis stages and associated with GO:0048232 descendant terms, are shown in Figure S3. Overall, many genes related to spermatogenesis are overexpressed in the male larvae, which confirms an early, larval onset of spermatogenesis.

Additionally, to identify potential upstream regulators, we searched for sex-determination transcription factors (*Dmrt*, *FoxL*, and *Sox*) among the annotated DGE. Two genes exhibit male-specific upregulation in the larval stages: *Dmrt1L* (Ojap_1477_c0_g2, Figures S4 and S5) and a *FoxQ2* paralog (Ojap_4497_c0_g1, Figures S4 and S6). *Dmrt1L* shows a male overexpression in the 2-day stage but only significantly in the juvenile and adult stages (Figure S4; Table S2). In contrast, we only find female-specific upregulation of the previously identified *Dmrt2*,^24^ significant only in the 5-day females (Ojap_1030_c1_g1, Figure S4). The *FoxQ2* paralog was initially annotated as *FoxL1* but is found in our orthology analysis to group with the annelid *Capitella teleta FoxQ2* and *Owenia fusiformis FoxQ2_11* (Figure S6). This *FoxQ2* is highly overexpressed in the males from the 2-day larval stage and significantly from the 3-day stage (adjusted *p* value < 0.01, Table S2; Figure S4). Additionally, we find late female-specific expression for *FoxL2* (orthology confirmed, Ojap_9080_c2_g1, Figures S4 and S6). The class expansion in spiralia, with 11 *FoxQ2* paralogs in *O. fusiformis*, six in *C. teleta*, and four in *D. gyrociliatus*,^44^ leaves the function of the many paralogs unstudied. We propose that both *Dmrt1L* and *FoxQ2* are involved in the male sexual determination pathway due to their male-specific and early expression.

### Somatic growth arrest and completion of spermatogenesis

**Figure 4:**
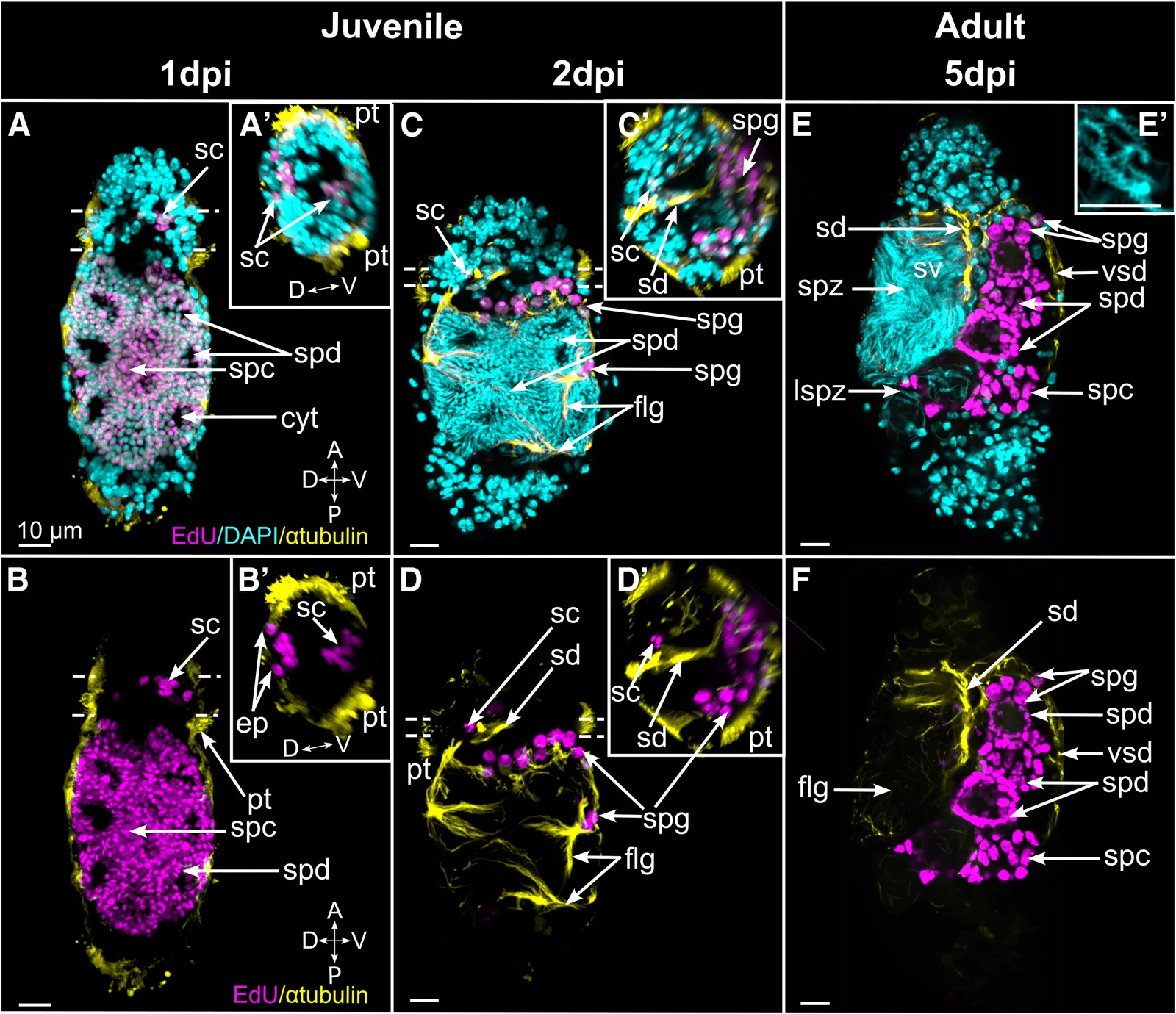
Post-metamorphic cell proliferation in males showing somatic growth arrest and advanced spermatogenesis (A–B′) 1-day post-induction (dpi) males display somatic and germ cell proliferation with advanced spermatogenesis, showing spermatocytes and early spermatids. (C–D′) 2-dpi males harbor late spermatids and proliferating spermatogonia and few anterior proliferative somatic cells. (E and F) 5-dpi males have mature spermatozoids contained in the seminal vesicle, show no sign of somatic cell division, and have started a second round of spermatogenesis with proliferating spermatogonia. CLSM section showing nuclei (cyan) incorporation of EdU (magenta) and acetylated alpha-tubulin-like immunoreactivity (LIR) (yellow). Dotted lines delimit the transverse section. Scale bars are 10 μm. A, anterior; cyt, cytophore; D, dorsal; ep, epidermis; flg, flagellum; lspz, loose spermatozoid; P, posterior; pt, prototroch; sc, somatic cell; sd, seminal duct; spc, spermatocyte; spd, spermatid; spg, spermatogonia; spz, spermatozoid; V, ventral; vsd, ventral seminal duct.

To confirm the somatic growth arrest in *Osedax japonicus* dwarf males, we visualized proliferating cells during post-metamorphic development and observed no dividing somatic cells in males after 5 days post-induction (dpi) (Figure 4F). In the metamorphosing males (1 dpi), only a few cells are EdU^+^: three to six somatic cells in the prototroch epidermis and seven elongated cells located more centrally in the anterior part (Figure 4B′). At this stage, males also lose the ventral and posterior ciliary rings (paratrochs 1 and 2 and telotroch) but retain an incomplete larval prototroch with a dorsal gap (Figures 4B′–4D′; Worsaae et al.^10^). The seminal duct is not yet visible. In 2-dpi juvenile males, few somatic cells remain proliferative around the U-shaped seminal duct (Figures 4D and 4D′), which is now formed and exits dorsally at the level of the prototroch gap (Figures 4C–4D′). At 5 dpi, males are fully mature, and only germ cells, identified by their morphology and restriction to the first segment, are found to be proliferative at that stage, confirming somatic growth arrest (Figures 4E and 4F). Now, males have a dorsal seminal vesicle formed by the enlargement of the dorsal part of the seminal duct (Figure 4E). This coincides with the presence of mature spermatozoids, showing the helicoidally spiraled nuclear shape characteristic of *Osedax*^15^^,^^20^ (Figure 4E′).

The changing proliferation pattern observed in males also reveals the specific timing of their progression through the stages of spermatogenesis. The late meiotic/early differentiation phases of spermatogenesis have already begun in 1-dpi males, with bundles of spermatocytes II showing EdU^+^ nuclei of smaller size lacking a cytophore (Figures 4A and 4B) and large clusters of EdU^+^ early spermatids, with small, round-shaped nuclei surrounding a central cytophore (Figures 4A and 4B). In the 2-dpi juvenile males, the first segment is mostly filled with EdU^−^ spermatids in the late differentiation phase, characterized by elongating nuclei oriented toward the center of the cluster and developing flagella oriented peripherally (Figures 4C and 4D). In the sexually mature adult males (5 dpi and older), the presence of both EdU^+^ spermatogonia and spermatocyte clusters (Figures 4E and 4F) in the entire first segment suggests that a second round of spermatogenesis is already taking place.

Female *O. japonicus* do not reach sexual maturity until 3 weeks post-induction of metamorphosis, and we observe a small number of EdU^+^ cells, located internally, in the epidermis and at the base of the forming dorsal palps in stages ranging from 1 to 5 dpi (Figure S7). This indicates continuous but slow somatic growth in females, post-metamorphosis.

### Heterochronic shift in gene expression between male and female

**Figure 5:**
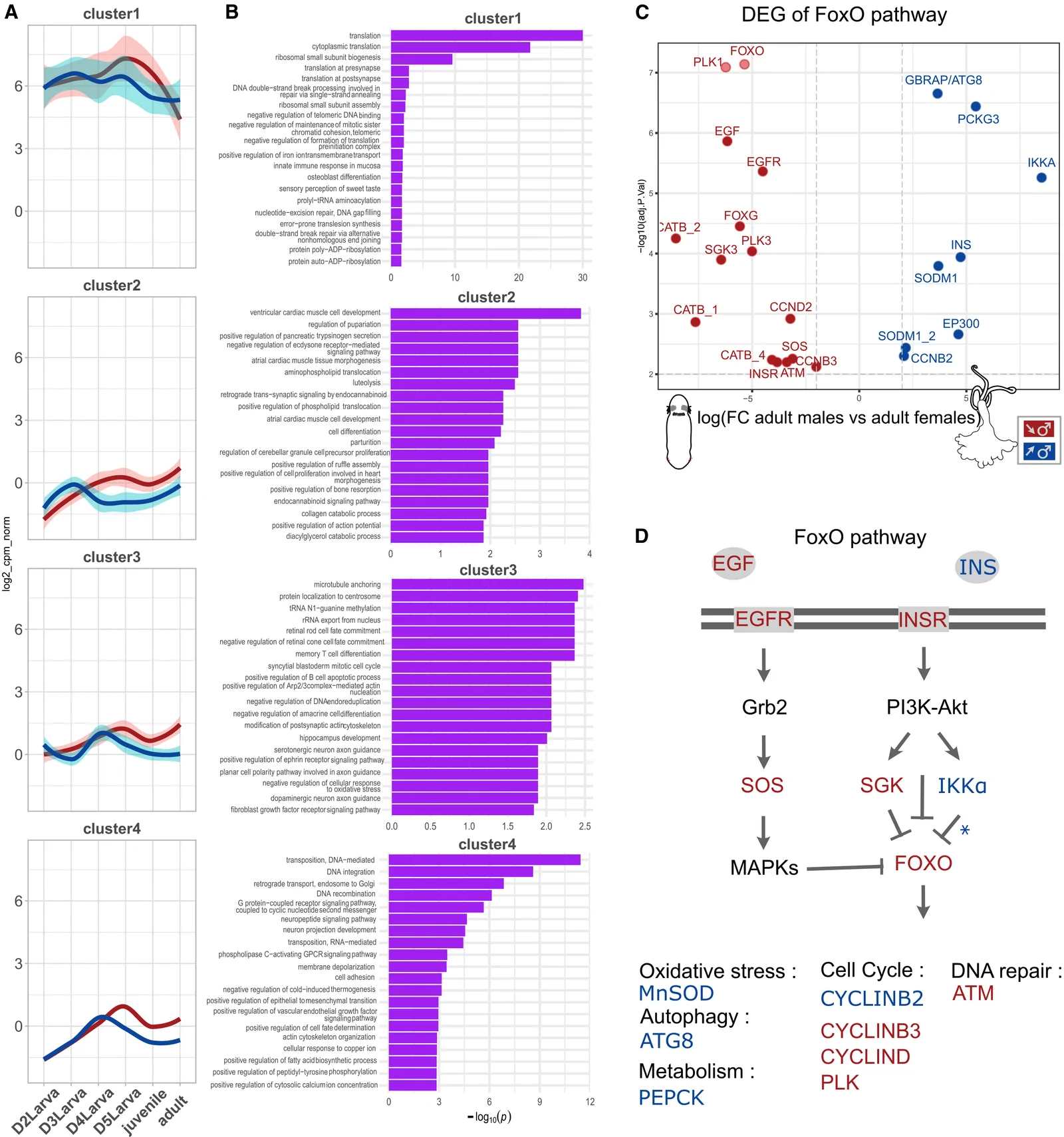
Heterochronic shifts to early offset of developmental processes in males (A) Subset of 4 out of 14 co-expression gene clusters (see also Figure S8) with early offset of expression in males vs. females (log-normalized cpm in female [red] and male [blue] from 2-day larval stage to the adult stage). Shading indicates the 95% confidence intervals. (B) Top 20 enriched GO terms in 4 selected clusters representing the −log10 (*p* value). (C) Volcano plot showing the FoxO pathway genes overexpressed (blue) and underexpressed (red) in the differential expression analysis of adult males versus adult females. (D) Scheme of the FoxO KEGG pathway displaying overexpressed (green) or underexpressed (red) genes in the adult males compared to the adult females.

Searching for gene expressions showing a temporal shift toward an early offset, we clustered the genes based on their temporal expression profiles in male larvae (2 to 5 days). Fourteen out of the 56 obtained clusters exhibit expression dynamics consistent with an early offset in males relative to females (Figures S8–S10). Of these, nine clusters (cluster 1, 2, 3, 7, 8, 12, 13, 14, and 16) exhibited a significant interaction between sex and stage (*p* value < 0.05; Table S4), supporting sex-specific expressions across development. Furthermore, eleven of the fourteen clusters (cluster 2, 4, 7, 9, 12, 13, 14, 15, 16, 19, and 28) were found to show a significantly higher eigengene values in the females at the 5-day stage, visually confirmed to support an earlier decrease in males (*p* value < 0.05; Table S4; Figure S11). We recovered three types of heterochronic patterns in males compared to females. The first pattern shows a similar onset of gene expression and an early offset in males. The second pattern exhibits a late onset coupled with an early offset of gene expression compared to females, likely reflecting accelerated developmental processes. The third pattern shows an early offset coupled with an early onset compared to females, therefore describing a process with same duration but an earlier shift in timing (for both onset and offset) (Figures 5A, S8, and S10). The heterochronic shifts encompass a wide range of GO biological processes, illustrating the diverse molecular mechanisms of male pedomorphosis (Table S4). Four of those clusters show enriched GO terms recognizably related to growth and somatic development as reflected in the pedomorphic phenotype (Figures 5A and 5B). For example, the cluster 1 is clearly dominated by genes annotated with GO terms associated with translation (Figure 5B). Cluster 2 includes terms involved in muscle development and cell differentiation (Figure 5B), while cluster 3 and 4 share genes associated with neurogenesis GO terms (e.g., neuron projection development, dopaminergic, and serotoninergic axon guidance) (Figure 5B). Moreover, in cluster 4, *FoxO*, a transcription factor involved in growth regulation and oxidative response, shows an early offset/downregulation in adult males compared to females (Figures 5B–5D, logFC = −5.4, *p* value < 0.01). Several other components of the FoxO pathway were differentially expressed between sexes. Eight genes, part of the FoxO Kyoto Encyclopedia of Genes and Genomes (KEGG) orthology, were downregulated in males, including the insulin receptor *INSR*, the *EGF* ligand and receptor, and the downstream cell cycle regulators *Plk*, *cyclinB*, and *cyclinD* or the DNA repair *ATM* (Figures 5C and 5D; Table S5). Furthermore, the inhibitor of FOXO, *IKK*α, is overexpressed in males (Figures 5C and 5D). The oxidative stress, autophagy, and metabolism components of the FoxO pathway do not appear to be affected by its downregulation (Figure 5C).

## Discussion

### Extremely early, larval onset of sexual maturation in *Osedax* males

To unveil the molecular basis of progenesis (heterochrony process), we studied the annelid *Osedax japonicus*, a dimorphic species with macroscopic females and miniaturized, microscopic males. With cell proliferation assays, *in situ* HCR of male gamete markers, and DGE analysis, we describe the genetic signatures of progenesis: how males undergo sexual maturation extremely early relative to females and cease to grow after metamorphosis, resulting in the approximately 100-fold difference in body size between the sexes. Our findings document the first example of a larval reproductive tissue (“larval testis”) in annelids. Even before metamorphosis in the 5-day-competent male larvae, cells labeled with the ciliary and male gamete marker *TBB* support the presence of specified male germ cells. Those *TBB*^+^ cells continue to divide and incorporate EdU between the 4- and 5-day larval stages. They resemble proliferating spermatogonia or even spermatocytes I or II with numerous, small, and round nuclei (Figure 2). This fast development is further corroborated by males 1 dpi of metamorphosis, already showing advanced stages of spermatogenesis with spermatocytes II dividing into early spermatids. By 2 dpi, males have entered late spermatogenesis showing characteristic late spermatid stages (Figures 4A–4D).

The male-specific larval expression of several genes associated with “male gamete formation” GO terms further support an extremely early onset of sexual maturation in *Osedax* male larvae. Genes involved in germ cell identity are upregulated at least from the 2-day larval stage; among them, a male-specific *Piwi* colocalized with *TBB* in mesodermal cells of male larvae (Figure S1), suggesting an early germline acquisition of sexual identity. Furthermore, the upregulation of *FoxQ2* and *Dmrt1L* confirms the acquisition of male identity necessary for an early onset of spermatogenesis, revealed by the expression of spermatogonia markers in later larval stages. Upregulation in the 4- and 5-day male larvae of genes involved in the maintenance or division of spermatocytes I, such as *RAE1* or *PCH2*, suggests the presence of not only spermatogonia but also actual spermatocytes in the 5-day male larvae (Figure 3J). Moreover, the transcription factor family *FoxJ* emerges as a promising candidate to investigate spermatocyte differentiation, with *FoxJ3* being overexpressed in *O. japonicus* juveniles (Figure 3J), while *FoxJ1 is* described as a spermatocyte II marker in *Mulina lateralis*^39^ and a testis-specific gene in *Patinopecten yessoensis*.^36^

Previous studies suggested a fast sexual maturation in male *Osedax* post-metamorphosis.^10^^,^^14^^,^^15^^,^^21^ We now reveal that this process starts already in the 4- and 5-day larval stages, before metamorphosis. Only the microscopic *Dimorphilus gyrociliatus* dwarf male^6^ has a similar fast male sexual maturation and short life span; however, this species lacks a larval stage. Sexual developmental processes beginning pre-metamorphosis in the larval stage and extending into adulthood were so far undocumented in annelids with a larval stage.^34^^,^^45^^,^^46^ Although the term “primary gonad” was used in the annelid *Platynereis dumerilii*, it described the non-proliferative PGCs rather than sexually differentiated gonads.^26^

The exceptional early onset of sexual maturation in *O. japonicus* males somewhat blurs the normally clear distinction between pre-metamorphic larval stages and post-metamorphic juveniles. This displacement of sexual maturation may be key to understanding progenesis. The heterochronic process, progenesis, is defined by an early offset of somatic growth, which has been proposed to be linked to or even initiated by an early sexual maturation, resulting in miniaturization.^1^^,^^2^^,^^3^^,^^11^ Most of the highly miniaturized lineages in Annelida (e.g., Diurodrilidae, Dinophilidae, and Nerillidae) lack a distinct larval stage,^47^ which may either have facilitated or be an outcome of progenesis. Although our study is limited by the absence of functional tools to test the causality between the two heterochronies by manipulating the delay of male sexual maturation, it is likely that the larval onset of sexual maturation in *O. japonicus* males also relates to their early arrest of somatic growth, microscopic size, and short lifespan.

### Early offset of somatic growth in male *Osedax*

We also demonstrate a lack of proliferating somatic cells in adult *O. japonicus* males, consistent with the early and synchronous offset of gross-morphological growth and rearrangements in nervous and muscular systems previously described in the dwarf males.^10^ In the adult males (5 dpi), only germ cells were consistently found to be proliferative. In the juvenile males (1 and 2 dpi), we found 10–13 proliferating cells not directly related to the germline (Figure 1). However, these cell divisions seemingly represent the developing seminal duct, which is developing here, at this stage. In the 5-day male larvae, only a few somatic cells around the dorsal organ were actively dividing, besides the proliferating germ cells. In contrast, many somatic cells were dividing in the female 5-day larvae and also in later female stages. Offset of somatic growth in *Osedax* males coincides with the completion of sexual maturation, although causality remains untested. We identified 14 clusters of genes with a progenesis signature, showing an early offset of expression levels in males compared to females, during development (Figure S8). The associated GO terms of the genes present in the 14 clusters, unveiled, e.g., an early cellular differentiation and translation, shifted to 3-day males versus 4-day females, potentially supporting precocious morphogenetic processes (Figures 5A and 5B). This suggests that progenesis in *Osedax* males is not only driven by shifts in sexual determination and maturation, as observed for the DMRT gene family, but also involves a broader acceleration of multiple different developmental programs. Notably, enriched GO terms associated with nervous system and brain development were associated to several clusters of genes with an early offset of expression, suggesting that neural differentiation may also be temporally advanced in males (Figures 5A and 5B). For instance, processes related to serotonergic and dopaminergic specification, appear to occur earlier in males (4-day larvae) than in females (5-day larvae) (Figure 5B).

The discovery of a pronounced downregulation of *FoxO* and multiple components of its signaling pathway in adult males points to a potential role of this downregulation in mediating somatic growth arrest during progenesis (Figure 5C). This early suppression of growth and maintenance pathways compared to the females is consistent with our results of somatic growth arrest in adult males and may also be reflected in their short life span (Figure 1).

Overall, our finding in *O. japonicus* provides genetic and cellular evidence of mechanisms underlying miniaturization through progenesis, extending our limited understanding of heterochrony in developmental and evolutionary processes.^6^^,^^7^ By revealing an early onset and acceleration of male sexual differentiation and gametogenesis coupled with an early arrest of somatic growth, our results confirm the theory of progenesis (heterochrony) as a central mechanism shaping miniaturization. Importantly, these shifts in developmental timing are not restricted to the reproductive function but extend to broader morphogenetic processes, including growth regulation and nervous system differentiation. Moreover, this work provides a mechanistic framework for understanding how heterochronic shifts can drive miniaturization and contribute to the evolutionary emergence of microscopic life forms from macroscopic ancestors, a process likely pervasive across metazoan evolution.

### Limitations of the study

The absence of an *O. japonicus* genome and functional tools limits this study and prevents us from confirming the role of *FoxQ2* in male sexual determination and experimentally testing the causality between the early sexual maturation and growth arrest.

## Resource availability

### Lead contact

Requests for further information and resources should be directed to and will be fulfilled by the lead contact, Katrine Worsaae (kworsaae@bio.ku.dk).

### Materials availability

This study did not generate new unique reagents or materials.

### Data and code availability

• Raw data comprising full confocal microscopy imaging scans are available in the ERDA archive, phylogenetic alignments are available on FigShare, and sequences used for transcriptomic analysis were retrieved from BioProject: PRJNA1088276 and PRJNA1217611.
• The code and related data are available through the GitHub repository.
• Additional information required to reanalyze the data reported in this paper is available in the supplemental tables.

## Acknowledgments

The authors would like to thank Ekin Tilic for interesting and insightful discussion on *Osedax* development and Rute da Fonseca for her valuable inputs on transcriptomic analysis. Funding from 10.13039/501100002808Carlsberg Foundation (CF15_0946 and CF20-0458), from Independent Research Fund Denmark (1026-00285B), and from the Ministry of Science and Higher Education Denmark (019200074B) was acquired by K.W. Funding from JSPS KAKENHI (18H04894 and 20 K06754) was acquired by N.M.

## Author contributions

Conceptualization, A.R., K.W., and N.M.; methodology, A.R., N.M., and K.W.; investigation, A.R., N.M., F.L.-R., and M.E.A.-R.; visualization, A.R. and K.W.; validation, A.R. and K.W.; funding acquisition, K.W. and N.M.; writing – original draft, A.R. and K.W.; writing – review and editing, A.R., M.E.A.-R., and K.W.

## Declaration of interests

The authors declare that they have no competing interests.

## STAR★Methods

### Key resources table

**Table:** 

REAGENT or RESOURCE	SOURCE	IDENTIFIER
**Antibodies**		

Mouse anti-acetylated α-tubulin	Sigma	RRID: AB_477585
goat anti-mouse Cy5	Jackson Immuno-Research	RRID: AB_2338716
goat anti-mouse 488	Jackson Immuno-Research	RRID: AB_2338854

**Chemicals, peptides, and recombinant proteins**		

DAPI-vectashield mounting media	VECTOR LABORATORIES	H-1200–10
paraformaldehyde (PFA)	ThermoScientific	043368.9M
PBS 10*X* UltraPure	VWR	K813
Tween 20	Millipore	655204
Magnesium Chloride (MgCl2)	Sigma Aldrich	M8266
Sucrose	Sigma Aldrich	S5390
Sodium Azide (NaN3)	Sigma Aldrich	S2002

**Critical commercial assays**		

HCR Buffers	Molecular Instrument	N/A
HCR™ Amplifier (v3.0) 488	Molecular Instrument	N/A
HCR™ Amplifier (v3.0) 546	Molecular Instrument	N/A
HCR™ Amplifier (v3.0) 647	Molecular Instrument	N/A
Click-iT Plus EdU Imaging kit Alexa Fluor™ 488 dye	(Invitrogen, Thermo Fisher Scientific	C10337

**Deposited data**		

*Osedax japonicus* transcriptome raw reads	NCBI BioProject	PRJNA1217611
*Osedax japonicus* transcriptome raw reads	NCBI BioProject	PRJNA1088276
Original code	GitHub repository	https://github.com/InvertCoding/Ojap_DwarfMale
Gene family phylogenetic data	FigShare repository	https://doi.org/10.6084/m9.figshare.31346782
Sequence available in Table S6	NCBI	Table S6

**Experimental models: Organisms/strains**		

*Osedax japonicus*	JAMSTEC Institute	N/A

**Oligonucleotides**		

HCR probe pairs	Custom/IDT pooled oligos	(Table S1)

**Software and algorithms**		

Imaris Viewer	Imaris Oxford Instruments	V10.0.0
R and Rstudio	R Development Core Team	v4.2.0
Inkscape	Inkscape Developers	V1.4.3
MAFFT	Katoh & Standley et al.^48^	v7
MUSCLE	Edgar et al.^49^	v3
trimAl	Capella-Gutiérrez et al.^50^	N/A
IQ-Tree	Nguyen et al.^51^	v3
ModelFinder	Kalyaanamoorthy et al.^52^	N/A
ultra-fast bootstrap	Hoang et al.^53^	N/A
FigTree	https://tree.bio.ed.ac.uk/software/figtree/	V1.4.4

### Experimental model and study participant details

We used a laboratory culture of the non-endangered marine annelid species, *Osedax japonicus* kept at JAMSTEC facilities. Females are kept in artificial sea water (ASW) at 10°C on vertebrate bones (see^18^^,^^21^). The developmental age was consistently reported along with the post-metamorphosis sex. The larval sex was assigned through the presence or absence of a gut, using confocal light scanning microscopy of acetylated α-tubulin like immunoreactivity. The influence of sex is the main topic of this paper and is described for various processes (see results).

### Method details

#### Experimental design

Our experimental design aimed at recording over time the cellular division in *Osedax japonicus* males to study the development of the gonads and the somatic growth arrest. We defined five critical stages, one pre-metamorphosis, i.e. the competent 5D larval stage, and four post-metamorphosis, comprising the very early metamorphosed 6 hours post induction, the early metamorphosed 1-day post induction (1dpi), the juvenile 2dpi and the mature adult 5dpi. Therefore, animals were treated with EdU (see below) 24h prior to the target stage. Both females and males were retrieved from those experiments, and their sex was assigned through the presence or absence of a gut, using confocal light scanning microscopy of acetylated α-tubulin like immunoreactivity.

#### Animal collection and fixation

Fertilized oocytes were sampled from the mucus surrounding the female palps and dissected out in filtered ASW. Time was registered from the first cleavage (hours post-cleavage (hpc)), and larvae, kept in 10°C filtered ASW conditions, were sampled at 1-day (1D=18+ hpc), 2-days (2D= 36+ hpc), 3-days (3D=60+ hpc), 4-days (4D= 84+ hpc) and 5-days (5D= 108+ hpc) larval stages. At the 5-day old stage, larvae are exposed to a sea bream scale (*Pagrus major*) previously washed and incubated in bacterial *Oceanospirillales* sp. culture to induce metamorphosis. Post-metamorphosis individuals were also kept in filtered ASW at 10°C and sampled days post induction (dpi) of metamorphosis.

#### Cell proliferation experiment and fixation

We used the Click-iT Plus EdU Imaging kit (Invitrogen, Thermo Fisher Scientific) to perform cell proliferation assay and followed the manufacturer protocol. We tested two incubation periods of 1h and 24h and performed our experiment with an incubation time of 24h. The stock solution of 10mM of EdU was prepared by adding 2mL of DMSO to the EdU and stored at -20C. For each stage (4D larval stage, 5D larval stage, 1dpi juvenile stage, 4dpi juvenile stage), 4uL of 10mM EdU stock solution was mixed in 4mL of filtered ASW to reach the manufacturer concentration of 10μM. Animals were incubated for 24h at 10°C before being anesthetized in 7% magnesium chloride (MgCl2) mixed 1:1 in filtered ASW. Samples were fixed in 4% paraformaldehyde (PFA) (ThermoScientific, 043368.9M) in phosphate-buffered saline solution (PBS) with 7% sucrose for one hour at room temperature (RT). Samples were rinsed in 7% sucrose PBS, six times before being stored with 0.05% NaN3 at 4°C.

#### Coupling of EdU, HCR and IF

Samples were washed for five minutes, three times in saline PBS containing 0.1% Tween20 (PBT). Samples were thereafter treated for (1) EdU/HCR/IF (Figure 2), (2) HCR/IF (Figure 3) or (3) EdU/IF (Figure 4), using an adapted version of the SHInE protocol.^54^ For protocol (1) and (2), samples were permeabilized for 30 minutes on ice in detergent buffer (0.5% Tween20, 1% SDS, 1mM EDTA (pH8), 150mM NaCl, 160mM Tris-HCl (pH7.5) in DepC_H2), followed by two PBT wash of five minutes on ice. Post-fixation was carried out for 1h at RT in 4% PFA on orbital shaking. Samples were rinsed in PBT four times for five minutes at RT, before being pre-incubated in 1:1 PBT/ hybridization buffer for 10 min at RT and pre-hybridize for 1h at 37°C in hybridization buffer (HCR-RNA-FISH buffers, Molecular Instrument). The samples were incubated with 8pmol of each probe set overnight at 37°C, followed by four washes of 15 minutes in hybridization wash buffer (HCR-RNA-FISH buffers, Molecular Instrument) and two washes of five minutes in 5*x* SSCT (5xSSC, 0.1% Tween20), and two times five minutes in PBT. For protocol (1) and (3), the Click reaction was performed following the manufacturer recommendations of the Click-iT Plus EdU Imaging kit (Invitrogen, Thermo Fisher Scientific). Samples were first permeabilized for five minutes in PBT containing 3% bovine seralbumine (BSA, Sigma-Aldrich 05470) before a 30 minute incubation at room temperature, in the dark, in freshly prepared Click reaction buffer. Briefly, we prepared 430 uL of fresh 1*X* reaction buffer from 10*X* stock ClickIT buffer, that we mixed, in order, with 20uL of CuSo4, 1.2uL of Alexa Fluor 488 and 50uL of freshly prepared 1*X* reaction buffer additive. For protocol (1) and (2), hairpin amplification step of HCR was performed by incubationg samples for 30 minutes at RT in amplification buffer (HCR-RNA-FISH buffers, Molecular Instrument). In the meantime, 15 pmol of each hairpin (h1, h2) for each (B1, B2, B3) amplifiers, was denatured for 90sec at 95°C and then cooled to RT for 30 minutes in the dark. Hairpins of amplifiers matching the probes were added to the amplifciation buffer. For protocol (1), (2) and (3), monoclonal mouse primary antibody against acetylated α-tubulin was added at this stage (1:800, SIGMA, T 6793). Hairpins and primary antibodies were incubated for 48 hours on a rocking table at 26°C protected from light. Afterwards samples were washed for 5 minutes two times, and two times for 30 minutes in 5*x* SSCT, followed by three washes of 10 minutes in PBT. Samples were incubated over 24h/48h with secondary antibody in PTA 0.1% (PBT, 0.25% BSA, 7% sucrose) (1:800, goat anti-mouse Cy5, Jackson Immuno-Research,115-175-062 or goat anti-mouse 488, Jackson Immuno-Research, 115-485-1460). Finally, samples were mounted individually after three washes in PBS 7% sucrose and transferred to DAPI-vectashield mounting media (VECTOR LABORATORIES, Burlingame, USA). Slides were kept at -20°C and scanned in the 2 weeks following mounting. Probes for *piwi1*, *nanos*, *DPCD*, *CENPF* and *CTSRE* did not show significant results (data not shown, Table S1).

#### Confocal imaging

The slides were scanned using a 60*X* lens on an OLYMPUS IX 81 inverted confocal microscope with a Fluoview FV-1000. Acquired z-stacks were analyzed with Imaris viewer 10.0 (Bitplane Scientific Software) and contrast and intensity were adjusted on the full z-stacks. Maximum intensity z-projections images were created from cropped or full z-stacks, specified in legends. Full scans used for the figures are available on an ERDA archive. Drawings and figures were made and assembled on Inkscape (v1.3.1).

#### Gene expression analysis

The transcriptome assembly and annotation used were previously described in.^24^ Here we performed a differential expression analysis between sexes per stage, comprising D2, D3, D4, D5, juvenile (1dpi) and adult (5dpi for males and 3weeks post induction for females) on R (v4.3.1) using Rstudio interface, using the *edgeR* package and Trimmed Mean of M-values (TMM) normalization method.^55^^,^^56^ Data were averaged based on developmental stage and sex, using the *limma* package.^57^ To complement the Trinotate KEGG annotation we extracted the amino acid sequence of significantly downregulated genes in adult males compared to females (logFC<|2|, *p*-value<0.01) and attributed KO identifiers using BlastKOALA.^58^ The gene clustering and co-expression analysis was performed using the *WGCNA* package^59^ and was based only on the larval male transcripts (2D-5D larvae), choosing an optimal soft thresholding power of 13. Block-wise network construction returned 56 modules displayed using all the transcripts (male and female) and module eigengenes were calculated on averaged expression per stage and per sex (Figures 5 and S8–S10). To assess differences in cluster expression between sexes across stages we performed a two-way analysis of variance (ANOVA), which was fitted using the module eigengenes calculated on individual replicates (Table S4) for each cluster using the following formula lm(ModuleEigengene ∼ sex ∗ stage), *p*-values were adjusted for multiple testing using the Benjamini-Hochberg method (Table S4). Pairwise comparisons between males and females estimated marginal means of eigengene were performed using the *emmeans* package along with a Holm correction for *p*-values adjustement (Table S4; Figure S11). Clusters showing a visual decrease in male expression, at least one stage earlier than females, with a significant combined effect of sex and stage or a significant difference in mean eigengene were kept, resulting in 14 clusters (Figures 5 and S8). Go-term enrichment of the “Biological Process” was performed using the *topGO* package^60^ we used a threshold of *p*-value < 0.05, calculated by a Fisher’s exact test. All the non-redundant Go-terms were fused by semantic similarities using the *simplifyEnrichment* package to ease visualization.^61^

#### Phylogenetics

Forkhead, Sox, and DMRT genes were identified from *O. japonicus* transcripts annotated with those PFAM families, with phylogenetic trees inferred from amino acid sequences using genes previously annotated in the literature,^33^^,^^44^^,^^62^^,^^63^^,^^64^ and others available on GenBank (Table S6). Sox and DMRT sequences were aligned with MAFFT^48^ using the einsi setting with a gap extension penalty set to 0, then trimmed with TRIM-Al^50^ with a gap threshold of 0.5, similarity threshold of 0.3 and consistency thresholds determined by the length of the aligned regions (Supplementary dataset). Forkhead sequences were aligned with MUSCLE^49^ with the default protein alignment setting, and the alignment was trimmed manually. Trees were inferred in IQ-Tree with ModelFinder and support was determined with 10,000 ultrafast bootstrap replicates.^51^^,^^52^^,^^53^ Trees were visualized in FigTree v1.4.4 [available from: https://tree.bio.ed.ac.uk/software/figtree/].

### Quantification and statistical analysis

For the transcriptomic analysis the read count per million were normalized using the Trimmed Mean of M-values (TMM) method, the differential expression was calculated between sexes of each developmental stage, and the Bayesian statistics were adjusted using the Benjamini-Hochberg (BH) correction (Table S4). We used the individual module eigengenes to perform a two-way analysis of variance (ANOVA) for each cluster using the following formula lm(ModuleEigengene ∼ sex ∗ stage), *p*-values were adjusted for multiple testing using the BH correction (Table S4). The *p*-values calculated from the estimated marginal means of individual eigengene between males and females of each developmental stage were adjusted with the Holm correction (Table S4; Figure S11).

## Supplemental information

Document S1. Figures S1–S11

Table S1. HCR probe target sequences and probe pair sequences, related to Figure 3

Table S2. GO terms related to male or female gamete formation genealogy, associated genes with a logFC > 2 in male larval samples, remaining “male gamete formation” GO term-associated genes, and female gamete formation-associated genes, related to Figure 3

Table S3. Differentially expressed genes/staged, related to Figure 3

Table S4. Linear model and eigengene mean comparison along with top 20 GO terms enriched in 14 clusters showing an early offset in males, related to Figures 5 and S11

Table S5. List of KEGG FOXO pathway and genes found over- or underexpressed in adult males compared with adult females, related to Figure 5

Table S6. Sequences of DMRT, Forkhead, and Sox families, related to Figures S5 and S6
