# Supplementary material for: Faster and smaller: Heterochronic shift in gene expression leads to larval onset of spermatogenesis and miniaturized males
Source: iScience. 2026 Sep 17;29(10):117511. doi: 10.1016/j.isci.2026.117511 (PMC13602260; doi:10.1016/j.isci.2026.117511)
Supplement: Supplementary file 1 [file mmc1.pdf]

iScience, Volume *29*

Supplemental information

Faster and smaller: Heterochronic shift in gene expression leads to larval onset of spermatogenesis and miniaturized males

Alice Rouan, Madeleine E. Aase-Remedios, Flora Lind-Rasmussen, Norio Miyamoto, and Katrine Worsaae

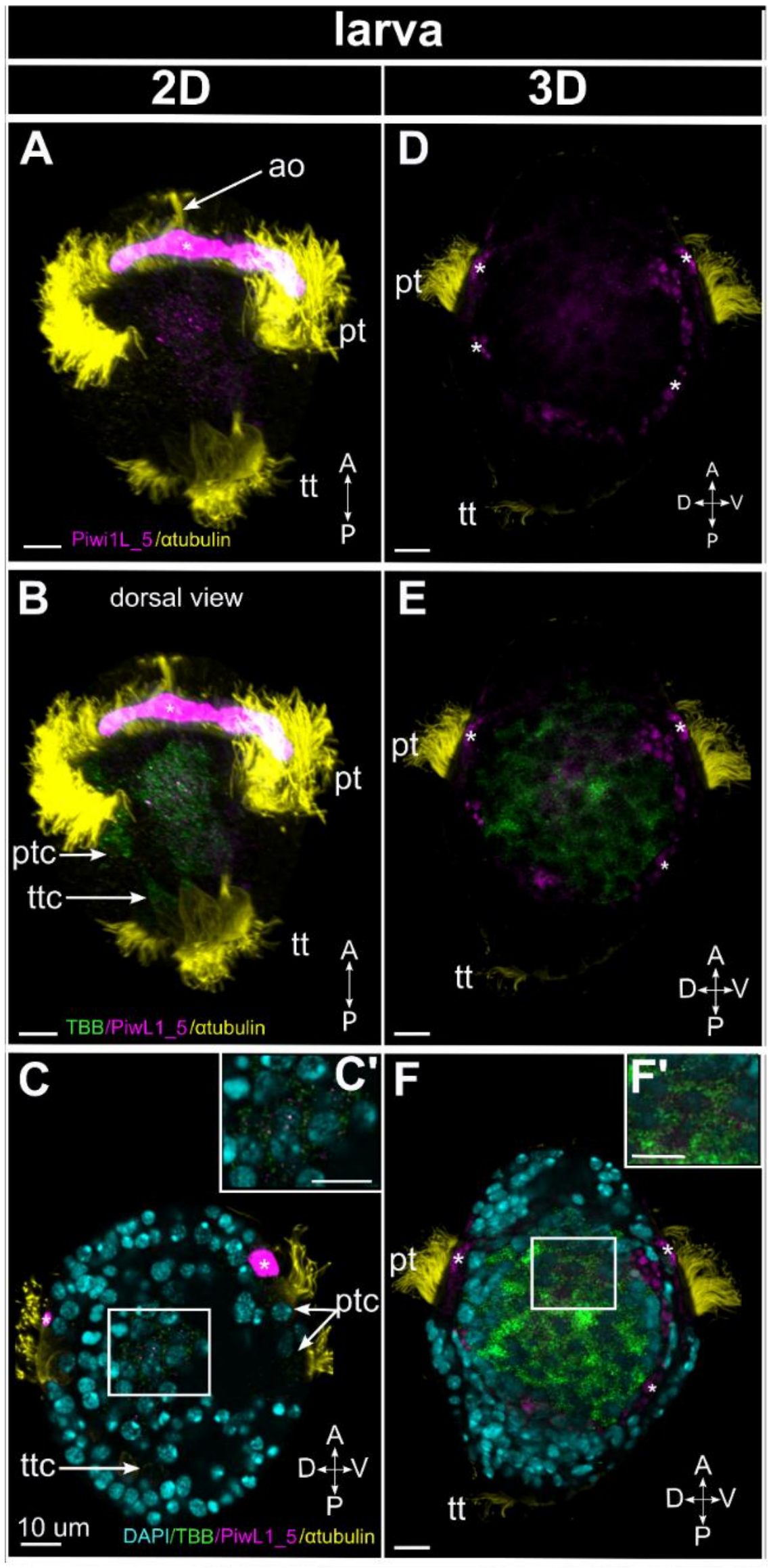

Figure S1. Temporal expression of *TBB* (green) and *PiwiL1_5* (magenta) coupled with nuclei staining (DAPI, cyan) and acetylated alpha-tubulin like-immunoreactivity (LIR) (yellow) in early larval stage. Related to Figure 2. (**A-B**) Maximum z-intensity projection (MIP) of male 2-days (2D) larva from a dorsal view. (**C-C'**) Middle MIP sections and inset showing the co-expression of *TBB* and *PiwiL1_5* in 2D male larva. (**D-F**) Lateral view of MIP middle sections showing mesodermal *TBB*/*PiwiL1_5* positive cells in a 3D male larva. A, anterior; ao, apical organ; D, dorsal; P, posterior; pt, prototroch; ptc, prototroch cell; tt, telotroch; ttc, telotroch cell; V, ventral; * prototroch glandular cell non-specific signal, yolk.

A Common male overexpressed genes

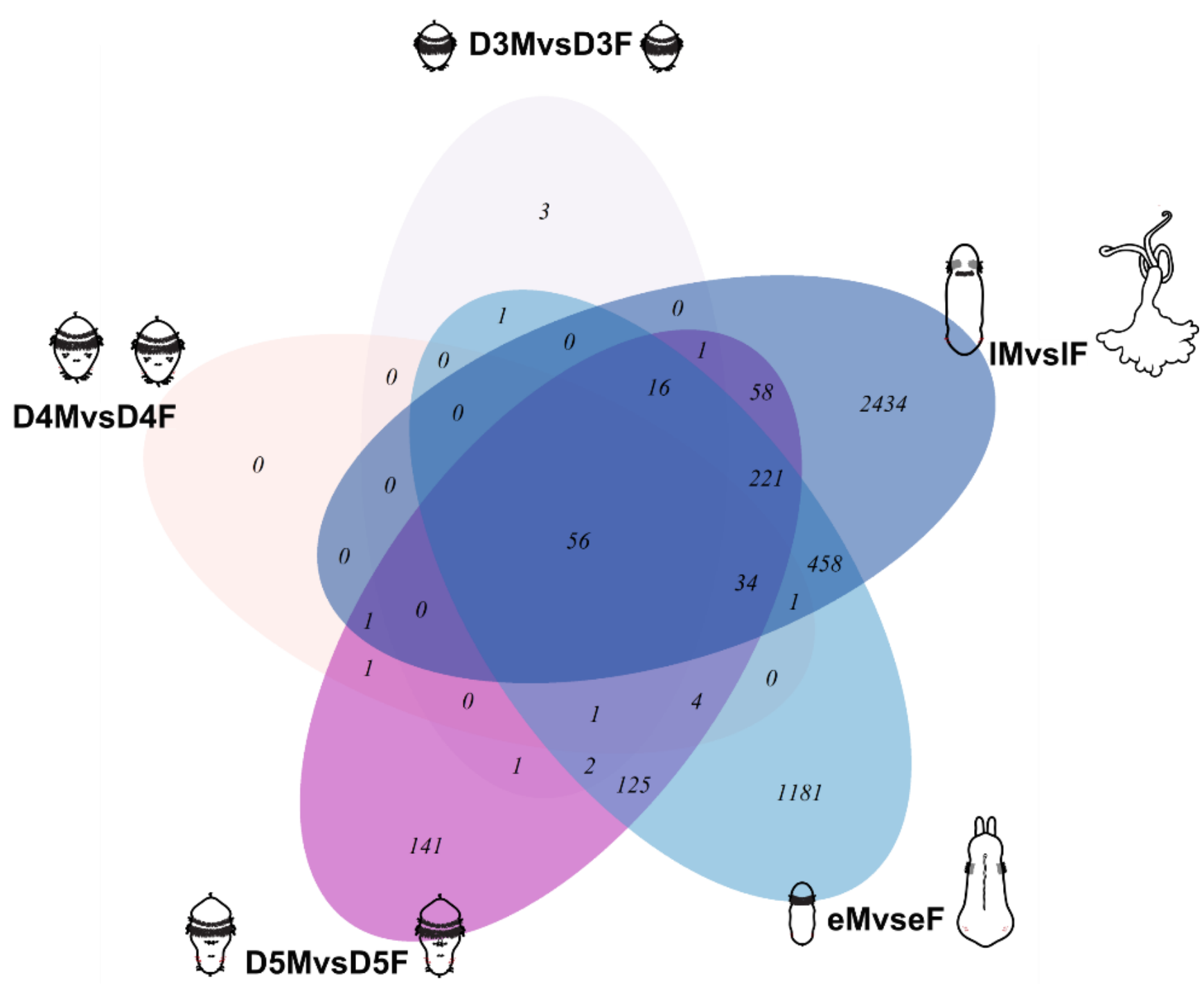

B Common male underexpressed genes

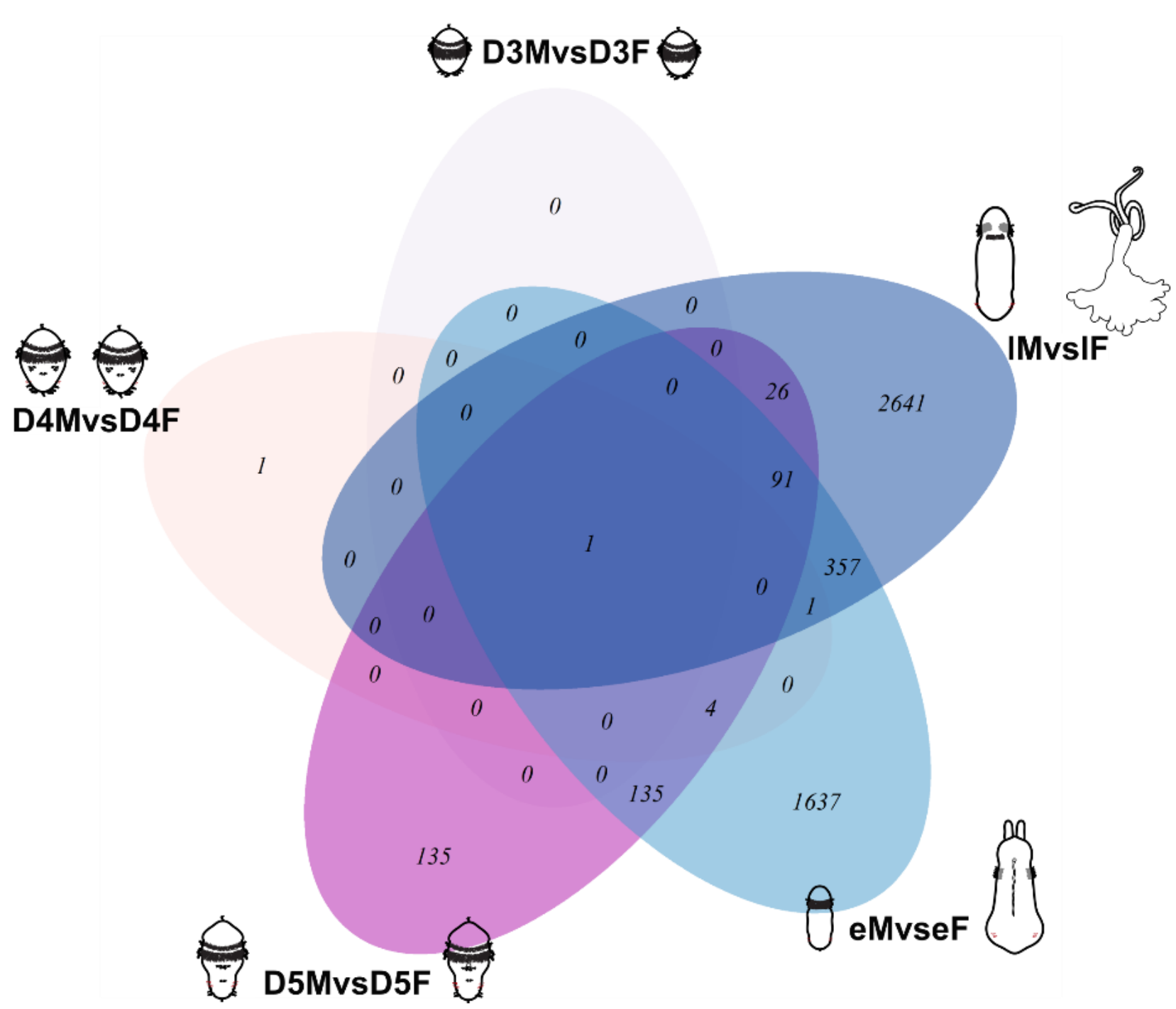

Figure S2. Venn diagram of (A) overexpressed genes in males compared to their female stage counterpart and (B) underexpressed. Related to Figure 3. Thresholds used were log(FoldChange) < |2| and adjusted p-value<0.01. D3, 3-days larva; e, early; F, female; l, late; M, male.

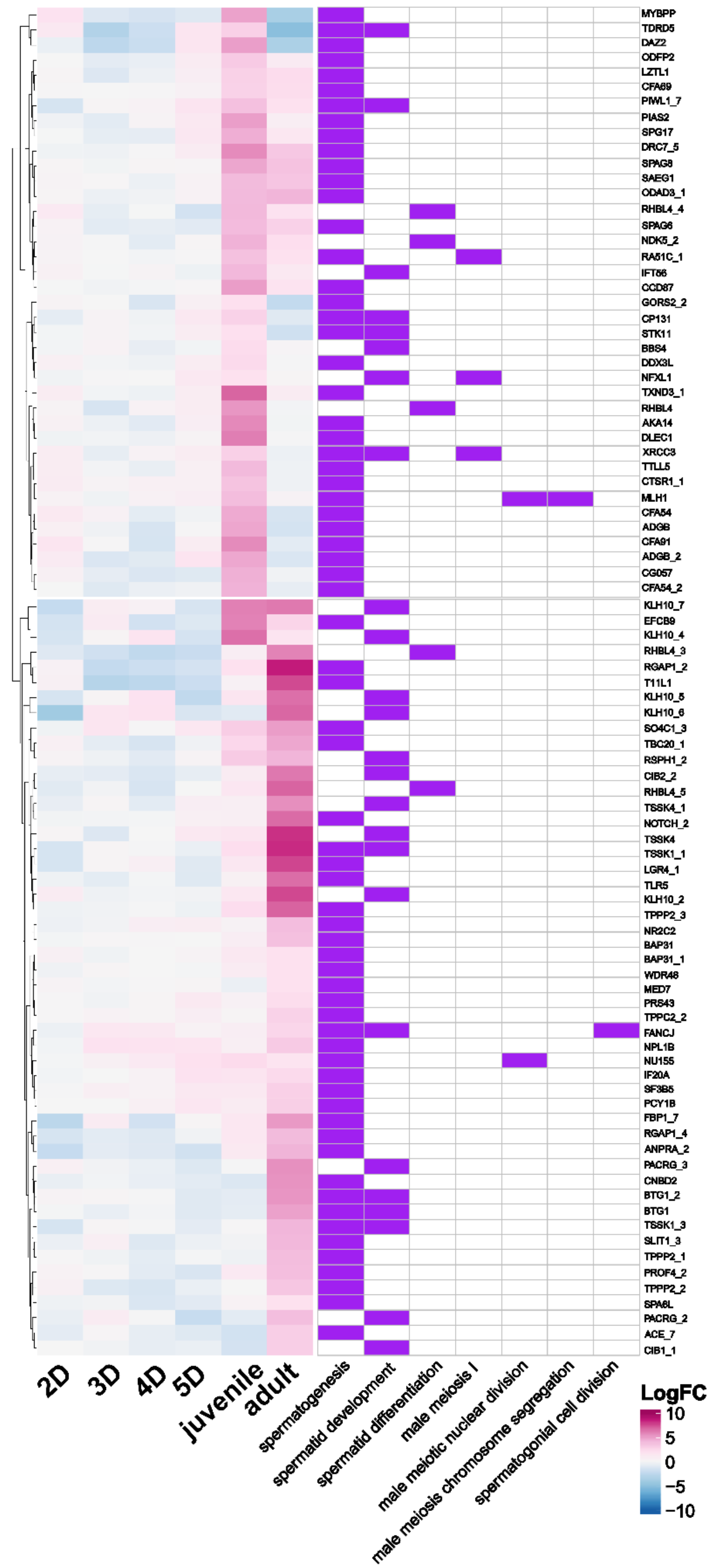

Figure S3. Heatmap of genes part of the GO term "male gamete formation" genealogy (purple), overexpressed in juvenile or adult males compared to their female stage counterpart. Related to Figure 3. Log(FoldChange) (logFC) of spermatogenesis genes overexpressed in juvenile and/or adult male stage (see Table S2).

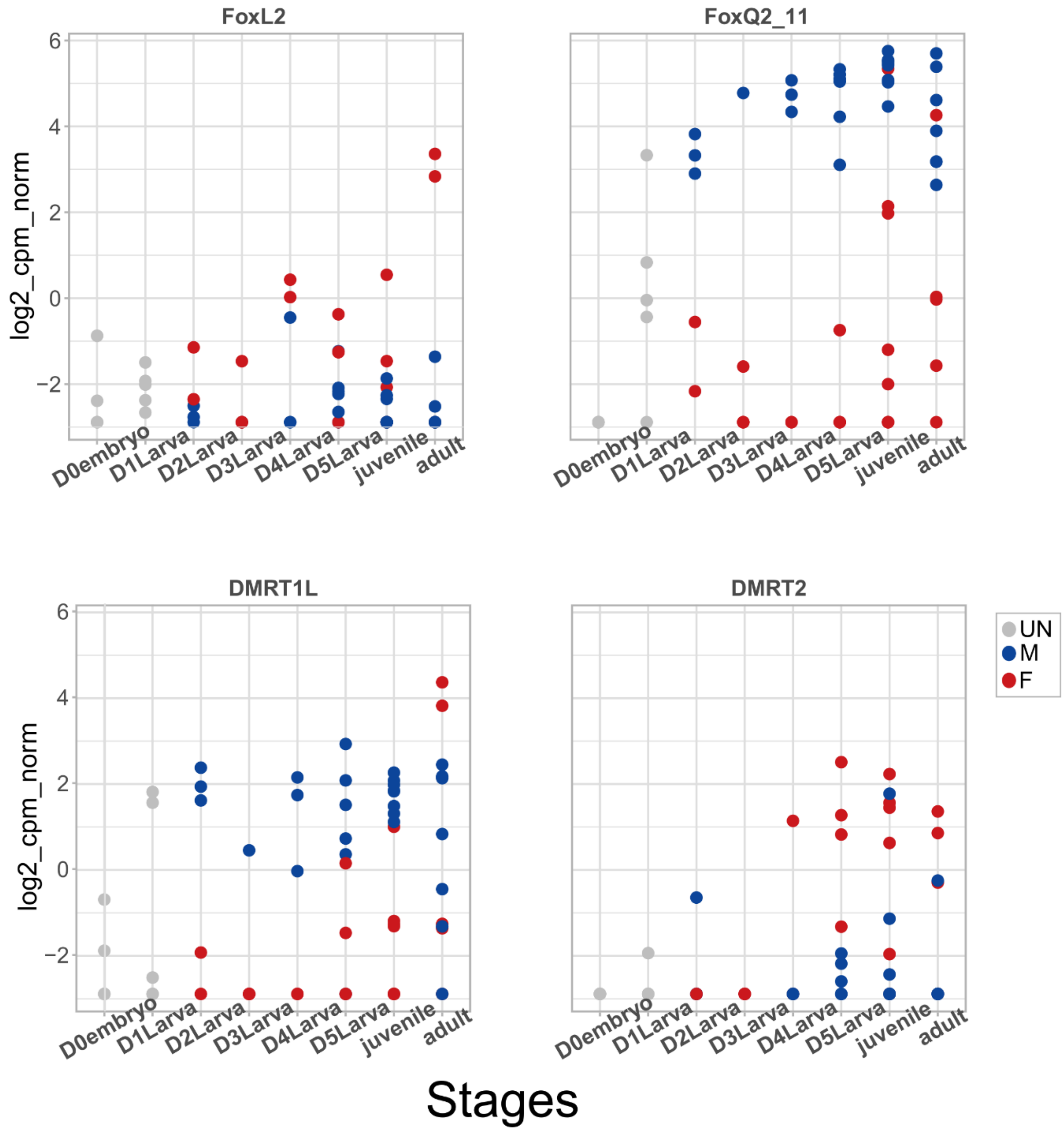

Figure S4. Expression of sex determination genes. Related to Figure 3. Individual gene expression (log2 of TMM normalized cpm) of temporal stages of *Osedax japonicus* (D0embryo-earlyembryo, 1D-5D larva, juvenile and adult) male (blue), female(red), and undetermined (grey) sex of *FoxL2* and *Dmrt2*, known female specific genes, and of *Dmrt1L*, known male determinant gene and the newly discovered *FoxQ2* paralog. *Dmrt1L* and *FoxQ2* show a male specific expression as early as D2Larva while *Dmrt2* and *FoxL2* are female specific and expressed later in development.

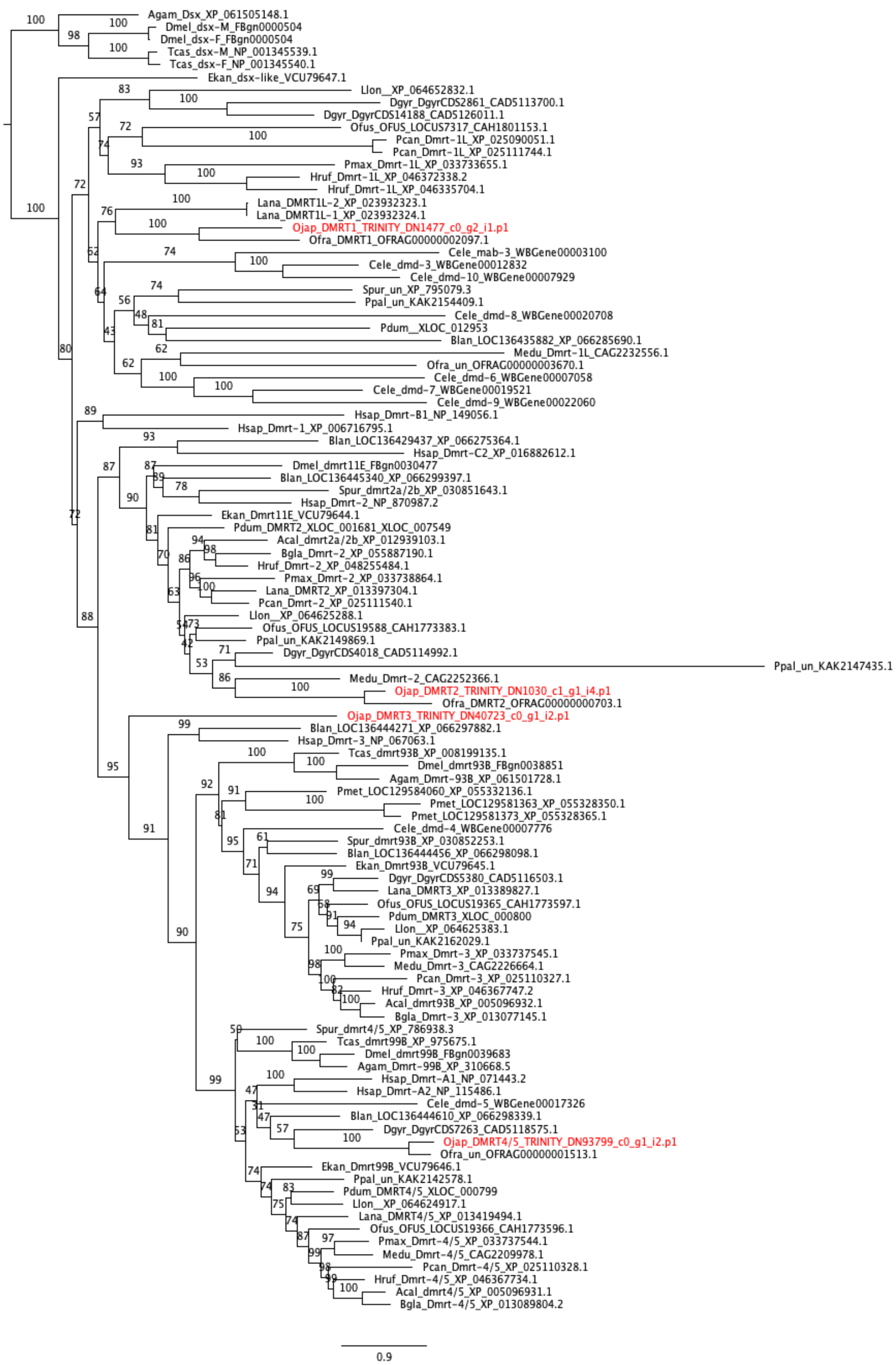

Figure S5. Maximum likelihood phylogeny of DMRT genes. Related to Figure 3. Branch labels show percent of 10,000 ultrafast bootstrap replicates. *O. japonicus* sequences are in red text. Species abbreviations: Acal: *Aplysia californica*; Agam: *Anopheles gambiae*; Bgla: *Biomphalaria glabrata*; Blan: *Branchiostoma lanceolatum*; Cele: *Caenorhabditis elegans*; Dgyr: *Dimorphilus gyrociliatus*; Dmel: *Drosophila melanogaster*; Ekan: *Euperipatoides kanangrensis*; Hruf: *Haliotis rufescens*; Hsap: *Homo sapiens*; Lana: *Lingula anatina*; Llon: *Lineus longissimus*; Medu: *Mytilus edulis*; Ofra: *Owenia frankpressi*; Ofus: *Owenia fusiformis*; Ojap: *Osedax japonicus*; Pcan: *Pomacea canaliculata*; Pdum: *Platynereis dumerilii*; Pmax: *Pecten maximus*; Pmet: *Paramacrobiotus metropolitanus*; Ppal: *Paralvinella* palmiformis; Spur: *Strongylocentrotus purpuratus*; Tcas: *Tribolium castaneum.*

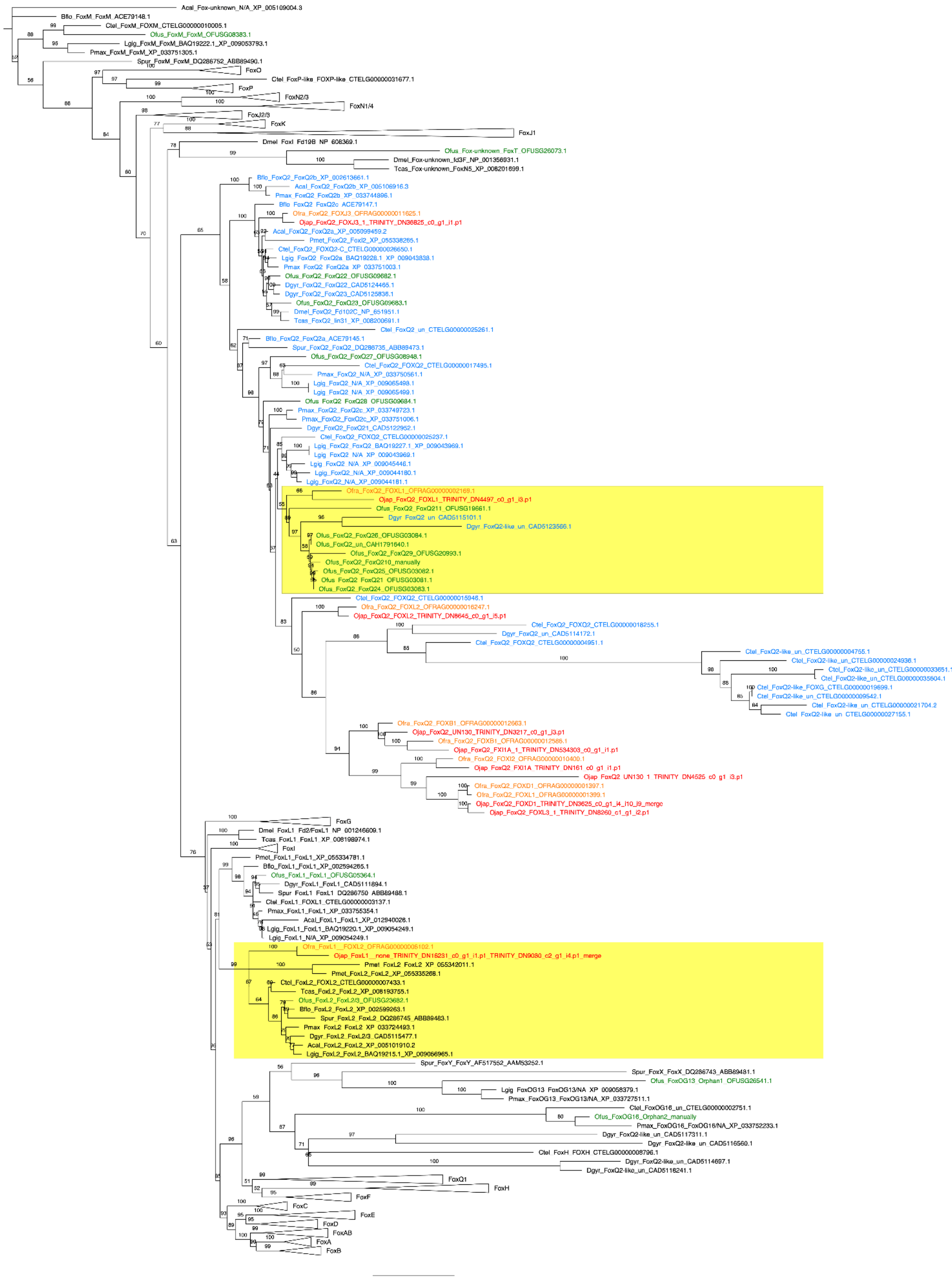

Figure S6. Maximum likelihood phylogeny of Forkhead-box (Fox) genes. Related to Figure 3.

Branch labels show percent of 10,000 ultrafast bootstrap replicates. *O. japonicus* sequences are in red text, *O. fusiformis* sequences are in green, *O. frankpressi* sequences are in orange, and *FoxQ2* genes from all other species are in blue. Yellow boxes highlight the clades including the differentially expressed *FoxQ2* paralog and *FoxL1* from *O. japonicus.* Sequence identifiers are identical to those in TabS6. Species abbreviations: Acal: *Aplysia californica*; Bflo: *Branchiostoma floridae*; Ctel: *Capitella teleta*; Dgyr: *Dimorphilus gyrociliatus*; Dmel: *Drosophila melanogaster*; Lgig: *Lottia gigantea*; Nvec: *Nematostella vectensis*; Ofra: *Owenia frankpressi*; Ofus: *Owenia fusiformis*; Ojap: *Osedax japonicus*; Pmax: *Pecten maximus*; Pmet: *Paramacrobiotus metropolitanus*; Spur: *Strongylocentrotus purpuratus*; Tcas: *Tribolium castaneum*.

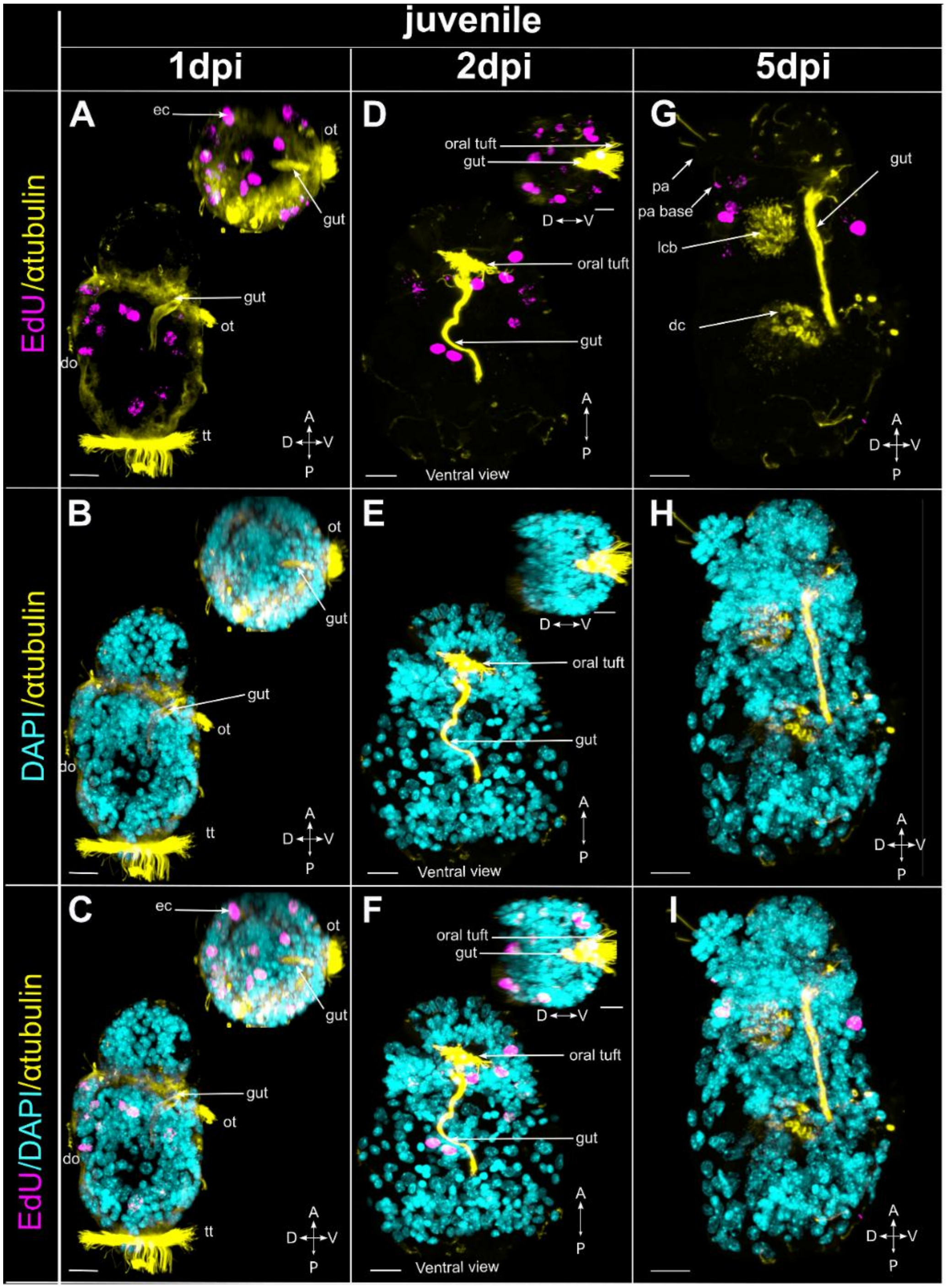

Figure S7. Cell proliferation assay showing nuclei (DAPI, cyan), incorporation of EdU (magenta), and acetylated α-tubulin like-immunoreactivity (LIR) (yellow) in post-metamorphosis females. Related to Figure 4. (A-C) Middle MIP sections of 1-day post induction (dpi) females display somatic cell proliferation in the epidermis, **(D-F)** Middle MIP sections of 2dpi females showing active somatic proliferation around the elongating gut and in the epidermis. **(G-I)** Middle MIP sections of 5dpi females show lateral somatic cell proliferation, notably at the base of the forming dorsal palps. Scale bars are 10µm. A, anterior; D, dorsal; dc, degenerated cilia; do, dorsal organ; ec, epidermal cell; lcb, lateral ciliary band; ot, oral tuft; pa, palp; P, posterior; tt, telotroch V, ventral.

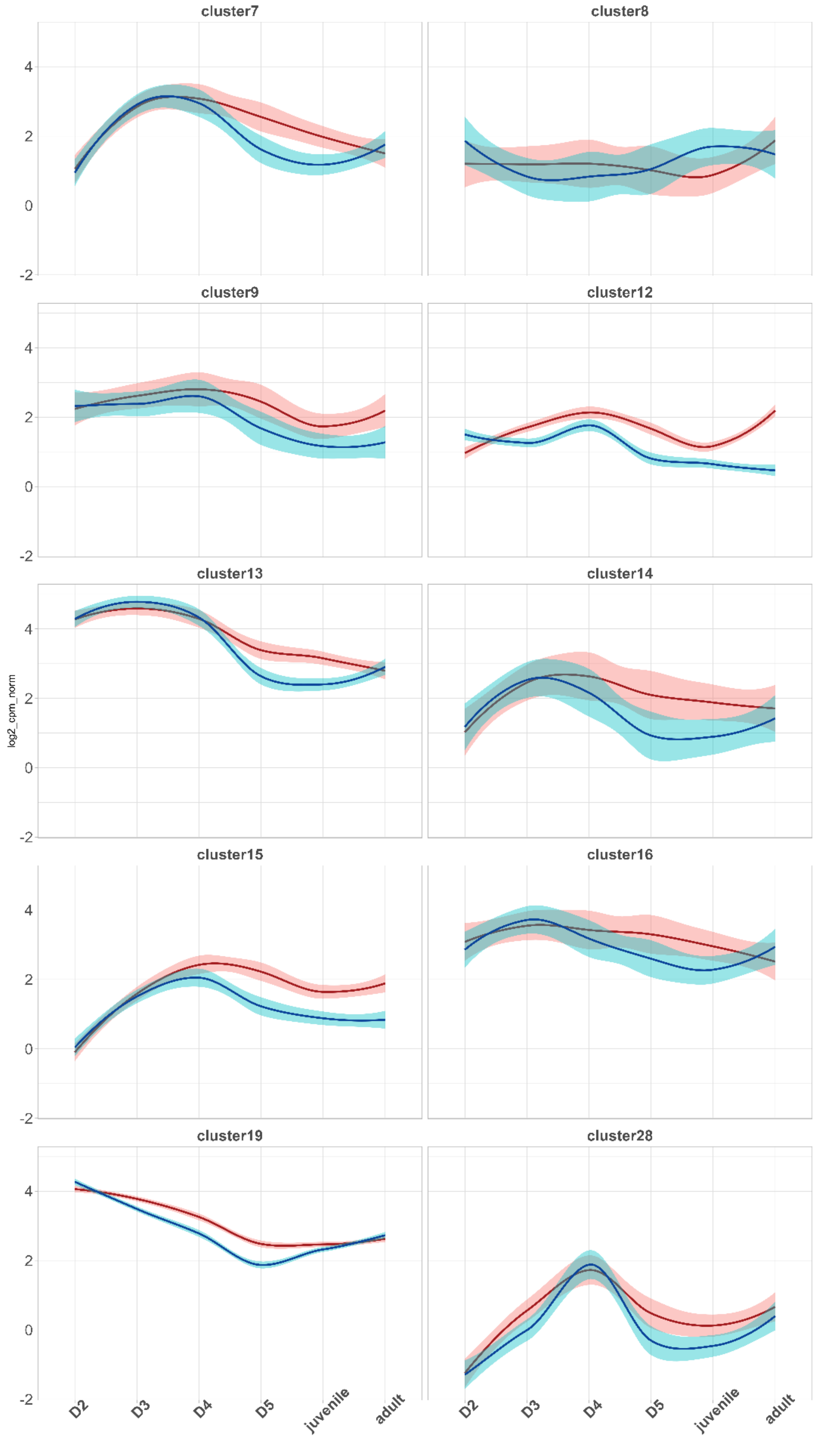

Figure S8. Clusters showing a statistically supported early offset of gene expression in males (blue) compared to females (red). Related to Figure 5. Gene expression (log2 of TMM normalized cpm) of temporal stages of *Osedax japonicus* (2D-5D larva, juvenile and adult) showing different patterns of heterechronies. Associated Gene Ontology top enirched list is available in Table S4.

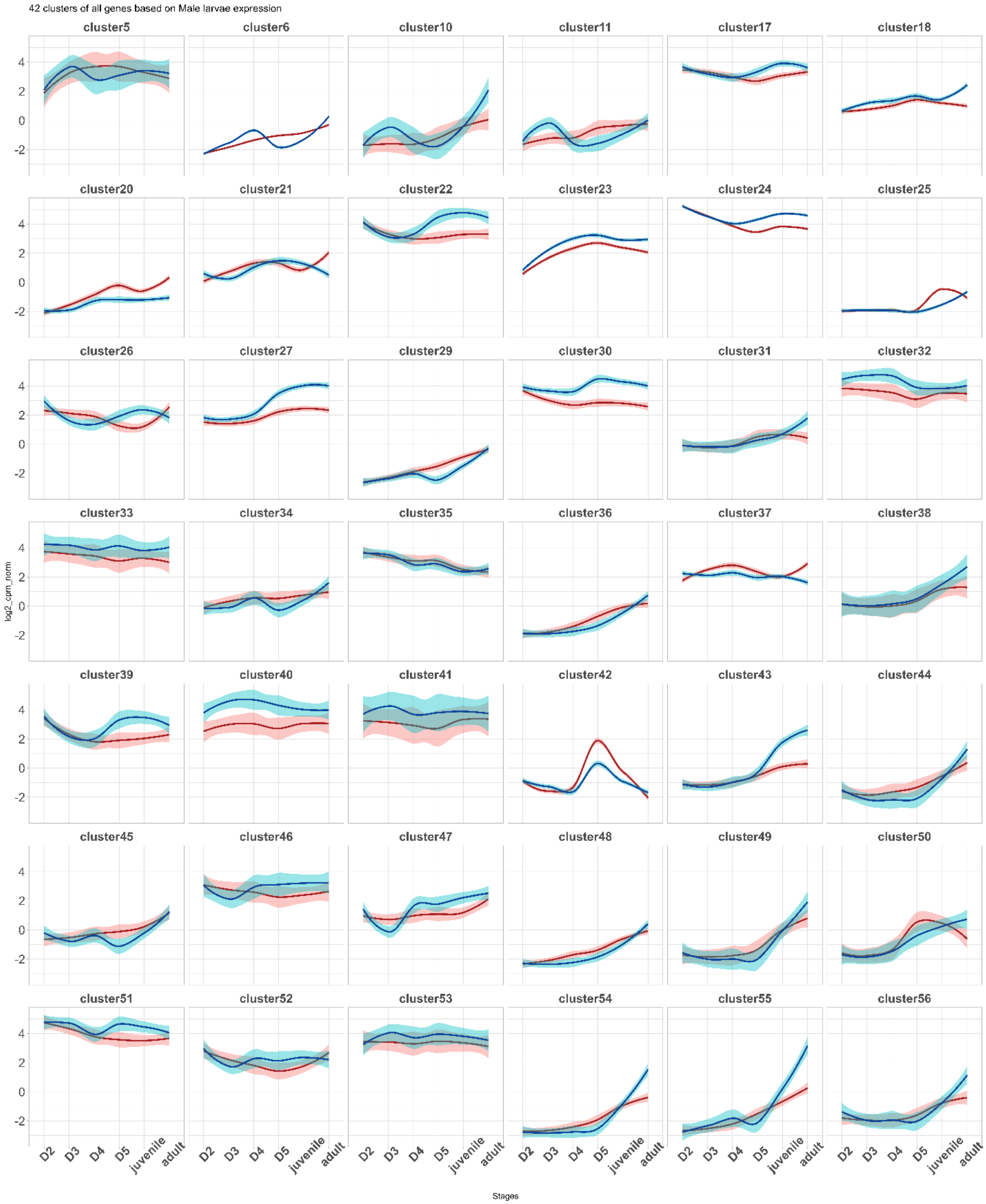

Figure S9. Clusters not showing a statistically supported early offset of gene expression in males (blue) compared to females (red). Related to Figure 5. Gene expression (log2 of TMM normalized cpm) of temporal stages of *Osedax japonicus* (2D-5D larva, juvenile and adult).

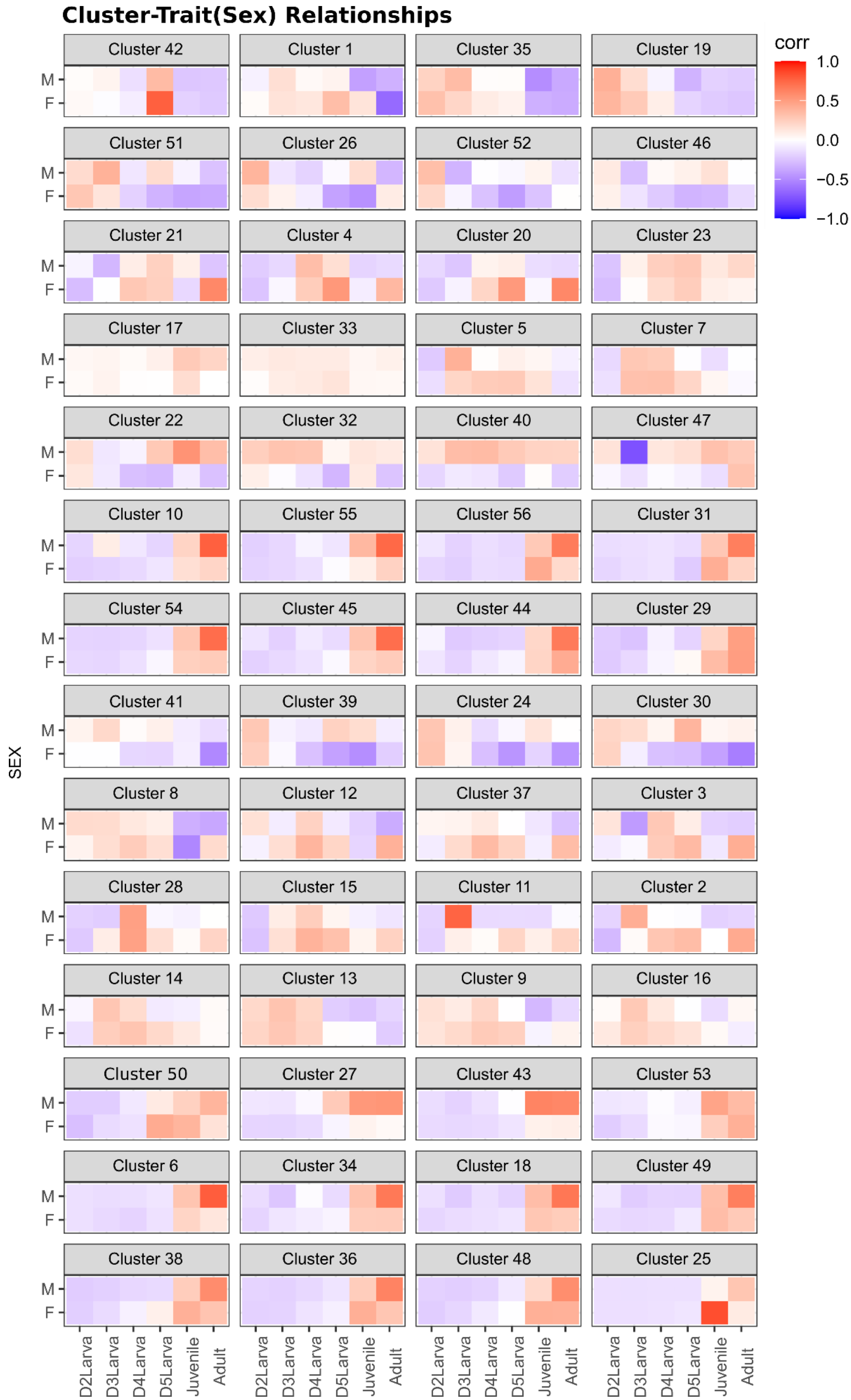

Figure S10. Cluster-trait correlation on averaged eigengene using sex (M=Male or F=Female) on temporal stages of *Osedax japonicus* (2D-5D larva, juvenile and adult). Related to Figure 5.

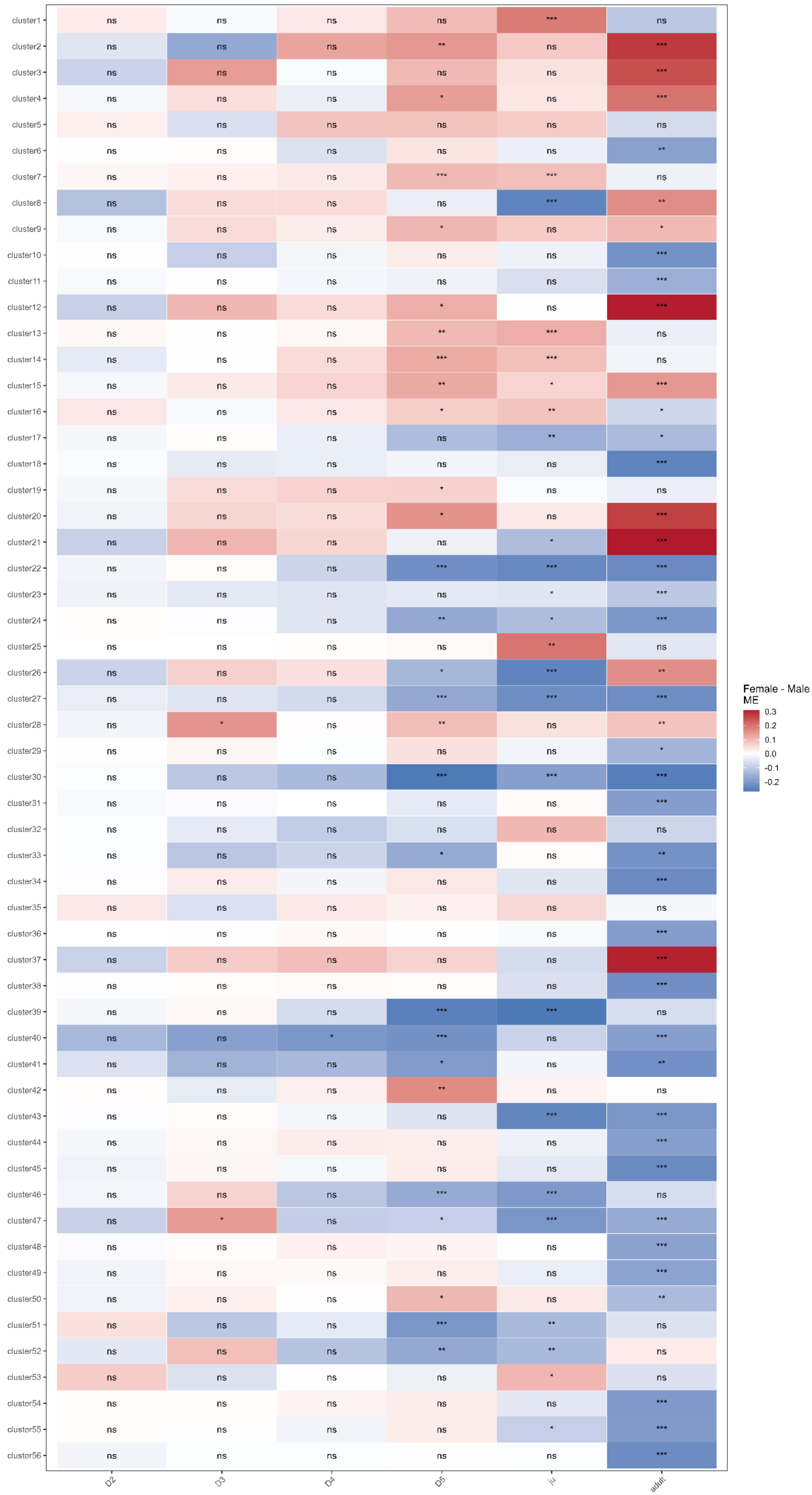

Figure S11. Heatmap of the Female-Male estimated marginal means calculated on the module eigengene (ME) per cluster on temporal stages of *Osedax japonicus* (2D-5D larva, juvenile (ju) and adult), showing significance (ns, not significant; *, p-value<0.05; **, p-value < 0.01; ***, p-value <0.001). Related to Figure 5 and Table S4.
